# Deciphering the pangenome of the shellfish pathogen Vibrio europaeus: evolutionary history and functional impact of core and accessory genes in aquaculture

**DOI:** 10.1099/mgen.0.001682

**Published:** 2026-06-04

**Authors:** Sergio Rodriguez, Diego Rey-Varela, Andrés Blanco-Hortas, Clara Martinez, Paulino Martínez, Marie-Agnès Travers, Juan L. Barja, Javier Dubert

**Affiliations:** 1 Aquatic One Health Research Center (iARCUS). Department of Microbiology and Parasitology. CIBUS bldg – Faculty of Biology, University of Santiago de Compostela, Santiago de Compostela, Spain; 2 Departament of Genetics, Faculty of Veterinary, University of Santiago de Compostela, Lugo, Spain; 3 Ifremer, ASIM, F-17390 La Tremblade, France; 4 IHPE, Université de Montpellier, CNRS, Ifremer, Université de Perpignan Via Domitia, Montpellier, France

**Keywords:** anti-phage defence systems, pangenome, shellfish aquaculture, *Vibrio europaeus*, vibriosis, virulence factors

## Abstract

*Vibrio europaeus* is an important pathogen in shellfish aquaculture, yet its genomic diversity remains poorly understood. Here, we present the first comprehensive analysis of the *V. europaeus* pangenome, integrating genomic data from all strains available to date (*n*=39) sequenced specifically for this study. These were isolated from different aquaculture facilities (shellfish hatcheries) associated with mass mollusc mortalities from different geographical locations, years and host species. Our findings revealed an open pangenome with 61% of the genes associated with the accessory genome that contribute to environmental and host adaptations. Phylogenomic analyses of the core-genome (39% of the pangenome size) allowed us to evaluate the evolutionary history and intraspecific diversity of *V. europaeus* and revealed that Spanish strains displayed a much lower genetic variability than French, Chilean or American strains, probably due to a monophyletic radiation event. Functional annotation of core and accessory genes revealed the key virulence factors of the species, while it also disclosed that these are located mainly in the core genes. The high number of anti-phage defence systems encoded in the accessory genome explained almost all the variability of the species. The results provide important insights into the evolutionary history and ecological versatility of *V. europaeus*, with potential implications for diagnostics, epidemiological surveillance and disease management strategies in aquaculture.

**Impact Statement**

This study presents the first comprehensive pangenome analysis of *Vibrio europaeus*, an emergent pathogen responsible for severe economic losses in shellfish aquaculture, the second most important sector of global aquaculture. Here, we characterized for the first time the *V. europaeus* pangenome, integrating genomic data from all strains isolated to date, sequenced specifically for this study using Next-Generation Sequencing (NGS; Illumina) and/or third-generation (PacBio) technologies. This work achieved the most complete species pangenome to date and is among the first studies on aquaculture-related bacterial pathogens. Beyond a descriptive framework, the pangenome was critically examined to identify key traits, including virulence factors, secondary metabolite biosynthesis and antimicrobial resistance genes, essential for host infection and adaptation. Moreover, the study of anti-phage defence systems was shown to account for much of the species’ genomic variability. The genomic resources and insights generated here substantially expand our understanding of *V. europaeus* biology and provide valuable information that can be applied to diagnostics, epidemiological surveillance and sustainable management of this pathogen in the aquaculture industry.

## Data Summary

All genome assemblies have been uploaded to the National Center for Biotechnology Information. The GenBank accession numbers for each of the 39 strains used in this study, along with detailed information, can be found in Table S1. All bioinformatics tools used for comparative genomics have been listed in the ‘Methods’ section, including references, associated databases and analysis parameters.

## Introduction

The term bacterial pangenome has been defined as the whole gene repertoire of a microbial species [1]. Advances in the study of the bacterial pangenome increase the understanding of the genomic adaptations of bacteria to the environment and contribute to the taxonomic and evolutionary research of the species [2,4]. The construction of a pangenome indeed reflects the diversity within a bacterial taxon and also enables the identification of common genes across strains or lineages, usually defined as the core genome. In opposition, the accessory genome would encompass the heterogeneity of genomes across strains or populations [1]. The core genome can be compared at various taxonomic levels, providing the genomic basis of the species phylogeny [1]. Additionally, the importance of the accessory genome relies on the fact that it enables bacterial adaptation to ecological niches, providing the variability underlying specific traits such as virulence, defence systems against bacteriophages, antimicrobial resistance or production of secondary metabolites [5,6]. Pangenome analyses are an excellent approach to studying pathogenic strains, facilitating the study of their adaptation to the environment and hosts.

Bivalve aquaculture is considered the second most important activity within the aquaculture sector, only surpassed by algae production [7]. However, its expansion is constrained by the negative impact of bacterial diseases, causing great economic and animal losses to the industry. One of the most prevalent diseases is vibriosis, caused by different pathogenic *Vibrio* spp. [8,9]. Although vibrios are commonly considered as opportunistic pathogens, some species from this genus are adapted to infect a variety of healthy hosts and bear virulence elements implied in different infection stages, such as bacterial adherence, invasion and survival in the host’s environment [10,13]. Among *Vibrio* pathogens in shellfish, *Vibrio europaeus* is an emergent species with great impact on bivalve aquaculture worldwide, affecting the most important mollusc species and infecting even different stages of development (i.e. broodstock, eggs, larvae, post-larvae and juveniles). *V. europaeus* has been identified in outbreaks in the main producer countries, such as Spain, France, Chile and the USA [9,14,19].

Pangenome analyses are key to obtaining insights into the species’ genomic diversity, with implications for understanding its evolution and adaptation and guiding applications such as secondary metabolite discovery and the development of preventive strategies. However, pangenome studies of bacterial pathogens affecting bivalve aquaculture are very scarce [20]. This study aimed to characterize for the first time the pangenome of the bivalve pathogen *V. europaeus* to gain knowledge about the genomics of this species. For this purpose, a collection of all *V. europaeus* strains isolated to date was sequenced and used to construct the bacterial pangenome. Core and accessory genes were identified, and different comparative analyses were performed to elucidate questions related to their evolution, intraspecific diversity, virulence, production of secondary metabolites and resistance. In a complementary study recently published by our group [21], the structural organisation of the accessory genome of *V. europaeus* was characterised through the identification of different mobile genetic elements (MGEs), and together these studies provide key insights into the evolutionary history and functional impact of the pangenome of this pathogenic species in aquaculture.

## Methods

### *V. europaeus* strains: DNA extraction, whole-genome sequencing and assembly

All strains identified to date as *V. europaeus* (39 strains) were used for the pangenome reconstruction, including 36 *V*. *europaeus* strains sequenced in this study and 3 genomes retrieved from the National Center for Biotechnology Information (NCBI) (strains 071316F, NPI-1 and CECT8426, Table 1). The 36 strains sequenced were obtained from six different hatcheries located between Spain (3), Chile (1) and France (2) and one environmental sample from the USA (Table 1). Two sequencing approaches were followed for genome assembly, as detailed in the workflow shown in Figure S1 (available in the online Supplementary Material): (i) short-read sequencing, 150 bp paired-end Illumina sequencing was performed in the *V. europaeus* strains (36 strains) for assembling at the scaffold level (Table 1). Strains were grown overnight in tryptone–casein soy agar supplemented with 2% (w/v) sodium chloride (TSA-2, Condalab) at 25 °C. Subsequently, a single colony was picked and grown under the same bacterial culture conditions in broth (TSB-2) with vigorous shaking. DNA was extracted from an aliquot of the overnight culture (200 µl) using the DNeasy Blood and Tissue Kit (QIAGEN) following the manufacturer’s instructions. Quantity, quality and integrity of each DNA extraction were evaluated using a NanoDrop One (Thermo Scientific), a Qubit (Thermo Scientific) and electrophoresis in a 1% (w/v) agarose gel. Genomic libraries and sequencing (HiSeq4000 sequencer, Illumina) were performed by the SNPsaurus company; (ii) long-read sequencing was performed in four representative strains (the type strain CECT8136 and the isolates CECT8427, PP2-843 and EX1, Table 1) to achieve high-resolution assemblies polished with Illumina reads from the previous section. For this, each bacterial strain was grown as described above, and HMW DNA was extracted from 4 ml of an overnight culture using the QIAGEN Genomic-tips 100 G^−1^ kit (QIAGEN). Quantity, quality and integrity of HMW DNA extractions were evaluated as described above. CECT8136, CECT8427 and PP2-843 strains were sequenced using a PacBio Sequel II sequencer (PacBio) by SNPsaurus, while the EX1 genome was sequenced using a MinION sequencer with the Rapid sequencing gDNA-barcoding kit (ONT). For both approaches, quality, assembly and polishing strategies are indicated on the workflow included in the Fig. S1. Genome completeness and contamination were assessed for all assemblies using CheckM [22]. Assembly quality and fragmentation were further evaluated using N50 and L50 metrics.

**Table 1.** Main features of all *V. europaeus* strains (39 strains) identified to date

Strain	Source	Location	Year	Mortality^†^	Reference^‡^
EX1	*O. edulis* larvae	Hatchery A (Galicia, Spain)	Jul/1985	Y	A
PP-654	*O. edulis* larvae	Hatchery B (Galicia, Spain)	Mar/2001	Y	B
PP-660	*O. edulis* larvae	Hatchery B (Galicia, Spain)	Mar/2001	Y	B
PP-635	*O. edulis* seawater tank	Hatchery B (Galicia, Spain)	Mar/2001	Y	B
CECT8136*	*O. edulis* seawater tank	Hatchery B (Galicia, Spain)	Mar/2001	Y	B
CECT8427*	*H. tuberculata* spat	Hatchery C (Normandy, France)	Jan/2004	Y	C,D
CECT8426*	*M. gigas* spat	Hatchery D (Nouvelle-Aquitaine, France)	Jun/2007	Y	C,D
07/038 2T2	*M. gigas* spat	Hatchery D (Nouvelle-Aquitaine, France)	Aug/2007	Y	C, D
07/108 T1	*M. gigas* spat	Hatchery D (Nouvelle-Aquitaine, France)	Aug/2007	Y	D
07/110 T1	*M. gigas* spat	Hatchery D (Nouvelle-Aquitaine, France)	Aug/2007	Y	C, D
07/112 T1	*M. gigas* spat	Hatchery D (Nouvelle-Aquitaine, France)	Aug/2007	Y	D
07/115 T2	*M. gigas* spat	Hatchery D (Nouvelle-Aquitaine, France)	Aug/2007	Y	–
07/116 T1	*M. gigas* spat	Hatchery D (Nouvelle-Aquitaine, France)	Aug/2007	Y	D
07/117 T1	*M. gigas* spat	Hatchery D (Nouvelle-Aquitaine, France)	Aug/2007	Y	C, D
07/120 T1	*M. gigas* spat	Hatchery D (Nouvelle-Aquitaine, France)	Aug/2007	Y	D
07/121 1T1	*M. gigas* spat	Hatchery D (Nouvelle-Aquitaine, France)	Aug/2007	Y	–
PP2-843	*R. philippinarum* spat	Hatchery E (Galicia, Spain)	Nov/2008	Y	B
PP2-978	*R. philippinarum* spat	Hatchery E (Galicia, Spain)	Nov/2008	Y	B
2909	*R. decussatus* seawater tank	Hatchery B (Galicia, Spain)	May/2011	Y	E
2895	*R. decussatus* seawater tank	Hatchery B (Galicia, Spain)	May/2011	Y	E
2930	*R. decussatus* larvae	Hatchery B (Galicia, Spain)	May/2011	Y	E
2951	*R. decussatus* larvae	Hatchery B (Galicia, Spain)	May/2011	Y	E
2945	*R. decussatus* seawater tank	Hatchery B (Galicia, Spain)	May/2011	Y	E
2967	*R. decussatus* larvae	Hatchery B (Galicia, Spain)	May/2011	Y	E
2968	*R. decussatus* seawater tank	Hatchery B (Galicia, Spain)	May/2011	Y	E
2969	*R. decussatus* larvae	Hatchery B (Galicia, Spain)	May/2011	Y	E
2971	*R. decussatus* seawater tank	Hatchery B (Galicia, Spain)	May/2011	Y	E
2974	*R. decussatus* larvae	Hatchery B (Galicia, Spain)	May/2011	Y	E
2975	*R. decussatus* seawater tank	Hatchery B (Galicia, Spain)	May/2011	Y	E
3454	*D. trunculus* larvae	Hatchery B (Galicia, Spain)	May/2012	Y	–
3492	*D. trunculus* seawater tank	Hatchery B (Galicia, Spain)	Jun/2012	Y	–
3610	*R. decussatus* broodstock	Hatchery B (Galicia, Spain)	Jul/2012	Y	F
3614	*R. decussatus* eggs	Hatchery B (Galicia, Spain)	Jul/2012	Y	F
NPI1	*A. purpuratus* larvae	Hatchery F (Coquimbo, Chile)	Feb/2015	Y	–
071316F	Seawater	Netarts Bay (Oregon, USA)	Jul/2016	N	G
L2	*P. rhomboides* larvae	Hatchery B (Galicia, Spain)	Mar/2018	Y	–
L3	*E. arcuatus* larvae	Hatchery B (Galicia, Spain)	Mar/2018	Y	–
L4	*E. arcuatus* larvae	Hatchery B (Galicia, Spain)	Mar/2018	Y	–
L20	*R. philippinarum* larvae	Hatchery B (Galicia, Spain)	May/2018	Y	–

*Synonymous names: *V. europaeus* CECT8136=PP-638 [17]; *V. europaeus* 04/002 1T2=CECT8427 and *V. europaeus* 07/118 T2 (CECT8426) [19].

†Strains isolated from mass mortalities (see references for more information).

‡References: A, Lodeiros et al. [91]; B, Prado et al. [17]; C, Saulnier [92]; D, Travers et al. [19]; E, Dubert et al. [15]; F, Dubert et al. [93]; G, Dubert et al.[18].

### Genome annotation and pangenome construction

Each genome assembly (39 genomes, Table 1) was annotated by Prokka 1.14.4 [23]. The GFF3 file was used to construct the *V. europaeus* pangenome using Roary 3.13.0 under default parameters [24]. The core genome includes those genes that were present in 100% of the *V. europaeus* genomes, while the accessory genome was further classified as shell core, soft core and cloud genome if a gene was present in 95–99%, 15–95% or 0–15% of the genomes, respectively. Genes classified into core, shell core, soft core or cloud fractions were plotted with the ggplot2 3.3.6 R package [25].

The R package micropan [26] was used to estimate the openness of the *V. europaeus* pangenome by calculating the Heaps’ law parameter (*α*), using 1,000 permutations. Results were plotted as described above. The application of Heaps’ law to genomic data has been described previously by [27]. Briefly, Heaps’ law was originally formulated for natural language corpora. In genomics, this is equivalent to saying that the rate at which new attributes (words or genes) are found decreases as one considers more and more entities (instance texts or genomes). That is, as sampling proceeds, discovering a new attribute becomes increasingly harder. Qualitatively, this is what we observe for the number of new genes discovered as one considers more and more genomes.

Gene sequences from each pangenome fraction (i.e. core, shell core, soft and cloud) were retrieved from Roary’s pan_genome_reference file using SeqKit 2.1.0 [28]. Subsequently, each gene was functionally annotated using reCOGnizer 1.7.0 [29] to obtain the distribution of Cluster of Orthologous Group (COG) categories within the *V. europaeus* pangenome. The percentage of genes assigned to the different COG categories per pangenome fraction was calculated, and the resulting matrix was plotted in a heatmap using the ComplexHeatmap 2.11.1 R package [30].

Identification of tRNAs and rRNAs from each genome assembly was done using tRNAscan-SE 2.0.1 [31] and barrnap 0.9 (https://github.com/tseemann/barrnap), respectively.

### Phylogenomic comparisons: phylogenetic tree, SNP identification and average nucleotide identity calculation

Genes from the core fraction of each genome assembly (39 genomes) were aligned by Roary 3.13.0 using MAFFT. This alignment was used to build a phylogenomic tree with IQ-TREE [32] based on the maximum-likelihood algorithm with the UNREST model by bootstrapping over 1,000 replications, and the phylogenomic tree was visualized in iTOL v6.6 [33].

SNPs were identified across the core genes from the alignment created with MAFFT using an ad hoc Python script (available at https://github.com/sergio-c-r/core-snps/blob/main/snp_dif.py). Average nucleotide identity (ANI) among the different *V. europaeus* genome assemblies was calculated by PYANI 0.2.11 [34] with default parameters and plotted in a heatmap as described in the ‘Genome annotation and pangenome construction’ section.

A dendrogram of the accessory gene profiles was constructed by hierarchical clustering using base R and a complete-linkage strategy, based on the presence/absence matrix generated by Roary. To illustrate the presence/absence gene profiles, the results were plotted as a heatmap using the R package ComplexHeatmap 2.11.1, including the dendrogram. Finally, principal component analysis (PCA) was performed in R on the binary pangenome gene presence/absence matrix using an Singular Value Decomposition (SVD)-based approach.

### Experimental infections

A total of 38 *V*. *europaeus* strains (71316F was not available in our bacterial collection, Table 1) were tested in virulence challenges using Manila clam (*Ruditapes philippinarum*) juveniles (14±1 mm). Virulence challenges were performed following the infection protocol described by Martínez [35]. *Vibrio breoganii* C5.5 was used as a negative control. Briefly, bacterial strains were grown overnight, and bacterial suspensions were made in sterile seawater adjusted to OD_600_=1 and confirmed by decimal dilution series onto TSA-2 plates (~10^8^ c.f.u. ml^–1^). Experimental challenges included two steps:

Infection: tanks were filled with filtered seawater (FSW) (0.22 µM Nalgene Rapid-Flow, Thermo Scientific) containing a bacterial suspension adjusted to a final concentration of 10^7^ c.f.u. ml^–1^. Subsequently, 15 Manila clam juveniles were added to each tank and kept for 24 h at room temperature (RT) (≈20 °C) for active bacteria filtration. Experimental challenges were performed in triplicate.

Post-infection: challenged juveniles were removed from the infection tanks and maintained for 8 h at RT to allow the internalization of the bacteria within the pallial cavity. Then, juveniles were transferred to new tanks filled with 200 ml FSW and maintained at RT with aeration. Mortalities were monitored at 8 h, 20 h, 32 h, 44 h, 56 h, 68 h and 80 h post-infection, and clams were immediately removed when the valves were open (dead juveniles) or siphons were not retracted following the stimulation (moribund juveniles). Results were recorded as a percentage of survival. FSW was renewed once per day (or early if it became turbid).

### *In silico* identification of the virulence genes

Genes related to virulence were identified in each *V. europaeus* genome (39 genomes) using the VFDB database [36]. An additional set of curated ad hoc database proteins whose role in virulence was experimentally demonstrated was included in the analyses by blastp comparisons. The resulting presence–absence matrix was used to plot a heatmap as described in the ‘Genome annotation and pangenome construction’ section. Subsequently, a PCA was performed using the prcomp function available in R base and plotted with ggplot2 to compare the virulence genes belonging to the non-core fractions among the different *V. europaeus* strains.

### Characterization of the antibiotic resistance profile

Antibiotic resistance profiles were obtained based on the consensus obtained between the *in silico* (i) and *in vitro* (ii) analyses and plotted in a presence–absence matrix using ComplexHeatmap 2.11.1:

The search for antibiotic resistance genes was performed using the web-based servers RGI 5.2.1 [37using the Comprehensive Antibiotic Research Database (CARD), considering only strict and perfect matches (bitscore >500) and ResFinder 4.1 [38] with an 80% threshold and minimum length.

Available bacterial strains (38 strains) were grown as described in the ‘*V. europaeus* strains: DNA extraction, whole-genome sequencing and assembly section, and antibiograms were carried out by the disc diffusion method on Mueller–Hinton agar (Oxoid) supplemented with 1% NaCl (MHA-1) with commercial discs (Oxoid, UK). The antibiotics tested were tetracycline (TE) (30 µg), oxytetracycline (OT, 30 µg), cephalexin (CN) (30 µg), ampicillin (AMP, 10 µg), amoxycillin (AML, 25 µg), erythromycin (E, 15 µg), enrofloxacin (ENR, 5 µg), flumequine (UB, 30 µg), cefoxitin (FOX, 30 µg), chloramphenicol (C, 30 µg), florfenicol (FFC, 30 µg), streptomycin (S, 10 µg), sulphonamide (SULDD, 25 µg) and gentamicin (CN, 10 µg). After incubation (24 h at 25 °C), the zones of inhibition around the discs were measured and compared against recognized zone size ranges established by the manufacturer for each specific antimicrobial agent.

### Search for secondary metabolite biosynthetic gene clusters

Biosynthetic gene clusters (BGCs) were identified in each bacterial genome (39 genomes) using the antiSMASH 7.0 web server (https://antismash.secondarymetabolites.org/) with default parameters and considering only strict findings. Genetic diversity within each BGC family among the 39 strain genomes was evaluated using BIG-SCAPE 1.1.5 [39], including the determination of biosynthetic gene cluster families (GCFs).

### Characterization of anti-phage defence systems

All genome assemblies (39 assemblies) were individually analysed using the following web-based servers: Defense-Finder [40] and PADLOC v1.4.0 [41] with the CRISPRDetect option [42]. A consensus between Defense-Finder and PADLOC outputs was shown with an absence–presence matrix, and a PCA was performed with each strain profile as described in the ‘Genome annotation and pangenome construction’ section.

## Results

### *V. europaeus* has an open pangenome dominated by the accessory genome

The features of *V. europaeus* assemblies used in this study are summarized in Table S1. Genome quality was evaluated using assembly statistics, including N50 and L50 values. As expected, fully resolved genomes showed the highest N50 values (>3 Mb) and the lowest L50 values (*n*=1), indicating very low fragmentation and very confident assembly quality. For the scaffold-level assemblies generated in this study, N50 values ranged from 329.6 kb to 1.5 Mb, while L50 values ranged from 1 to 4, with the number of contigs ranging between 32 and 47. Notably, the most fragmented assembly corresponded to the strain 071316F, which was not sequenced in this study due to the unavailability of the bacterial strain and was retrieved directly from the NCBI. In addition, genome quality was also assessed based on estimates of completeness and contamination. All genomes showed low levels of contamination (<2%) and high completeness, ranging from 98.7 to 100%

**Fig. 1.**
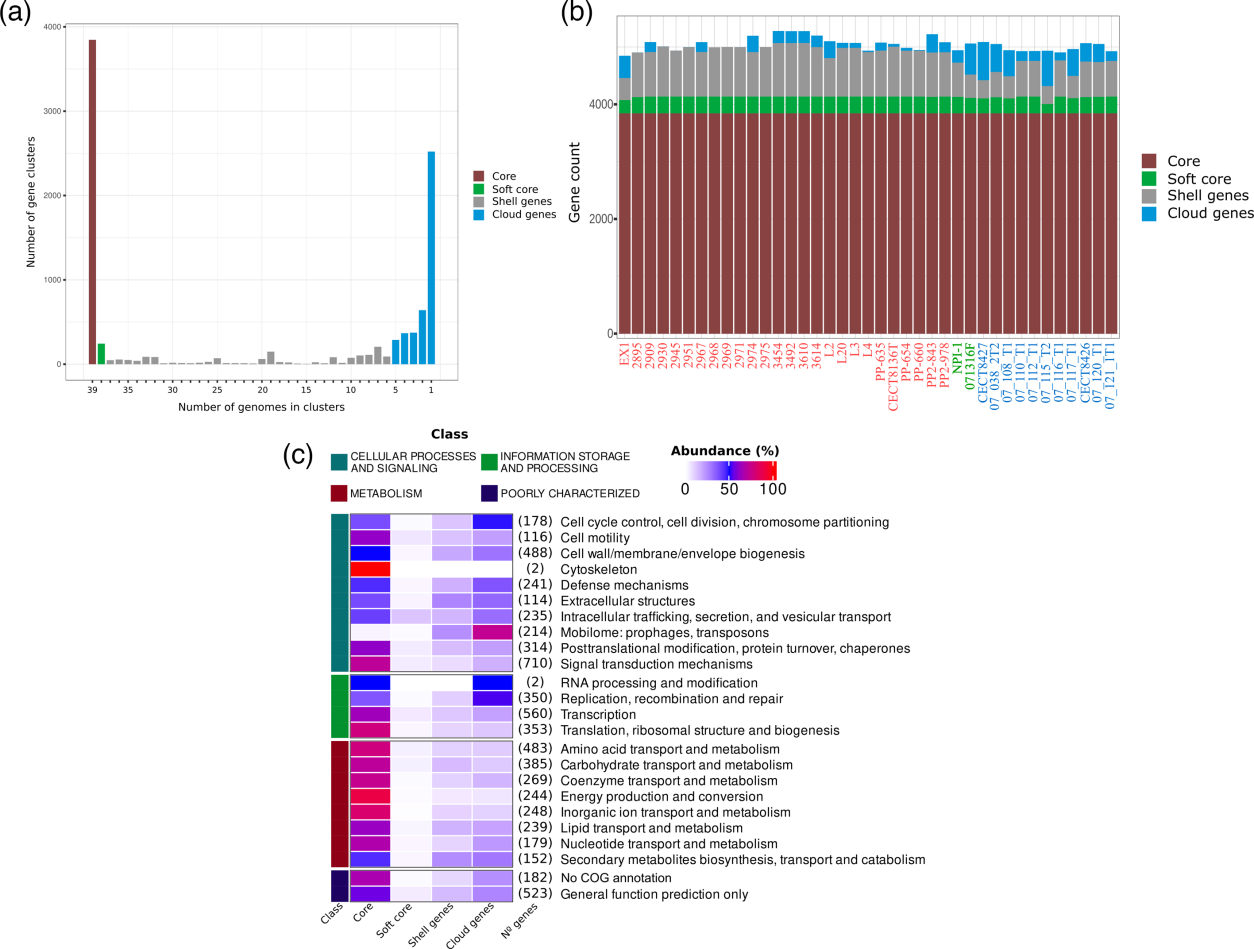
Main features of the *V. europaeus* pangenome. (**a**) Gene fraction (core, shell core, soft core and cloud) profiles. (**b**) Frequency distribution of gene clusters throughout the pangenome; colour of the strains indicates the origin: green indicates American strains, blue for French strains and red for Spanish strains. (**c**) Percentage of genes related to COG general functions found in core, soft core, shell core and cloud fractions of the pangenome. To facilitate comparison among fractions with different gene counts, the distribution in **(c)** is shown as percentages for each COG category distributed between each pangenome fraction.

The *V. europaeus* pangenome was composed of a total of 9,860 genes, and it is considered an open genome according to the Heaps value obtained (*α*=0.68) (Fig. S2). Among these genes, 39% (3,846 genes) were assigned to the core genome, whereas 61% (6,014 genes) belonged to the accessory genome. Within the accessory genome, the 26%, 4% and 70% were assigned to the shell, soft and cloud gene fractions, respectively (Fig. 1a and b). In relation to the geographical origin, the main source of cloud genes was derived mainly from the French strains (CECT8427=666 genes, 07/115 T2=617 genes, 07/038 2T2=487 genes, 07/117 T1=468 genes, 07 108 T1=455 genes, CECT8426=321 genes and 07/120 T1=316 genes), despite most of them being isolated from the same sampling time/location (Fig. 1b). A significant number of cloud genes were also associated with two Spanish strains (EX1=388 genes and PP2-843=320 genes) and with the American strain 071316F (542 genes) (Fig. 1b).

The 69% of the pangenome (6,781 genes) was accurately assigned to specific COG functions, representing 61%, 3%, 12% and 24% of the core, shell core, soft core and cloud fractions, respectively (Fig. 1c). For the majority of the COG categories established, the genes identified were located in the core genome category (>50%) (Fig. 1c). Interestingly, the COG functions in which accessory genes were predominant (>50%) were always classified into the cloud category, while they were almost absent in soft-core and shell genes. These more abundant categories in cloud genes were ‘Cell cycle control, cell division, chromosome partitioning’, ‘Defence mechanisms’, ‘Extracellular structures’, ‘Intracellular trafficking, secretion and vesicular transport’, ‘Mobilome: prophages, transposons’, ‘Replication, recombination and repair’ and ‘Secondary metabolites biosynthesis, transport and catabolism’ (Fig. 1c). Genes without COG annotation were detected across all pangenome fractions, with a higher relative proportion from the core, cloud and shell fractions.

Interestingly, a difference in the abundance of structural RNAs (tRNA and rRNA) was found between the six long-read sequencing assemblies and those sequenced with short-read Illumina technology. Among the Illumina assemblies, tRNAs ranged from 88 (071316F) to 109 (2971 and 97/121 1T1) (Table S2) with an average of 106 tRNAs/genome. However, the six highly contiguous chromosome-level assembled genomes encoded a higher number of tRNAs, ranging from 118 tRNAs (CECT8136 and NPI-1) to 122 tRNAs (CECT8427). Furthermore, rRNAs from Illumina assemblies displayed a lower copy number (1 copy for 16S rRNA, 1–4 copies for 5S rRNA and 1–2 copies for 23S rRNA) than the fully resolved genomes (9–10 copies for 16S rRNA, 10–11 copies for 5S rRNA and 9–10 copies for 23S rRNA) (Table S3). The six chromosome-level assembled genomes showed the highest number of RNA coding genes, probably due to the limitations of short reads to identify different genes of the same family, especially if they are tandemly arranged [43].

### Nucleotide diversity and phylogenomic analyses based on the core genome shed light on the evolutionary history and intraspecific diversity of *V. europaeus*

**Fig. 2.**
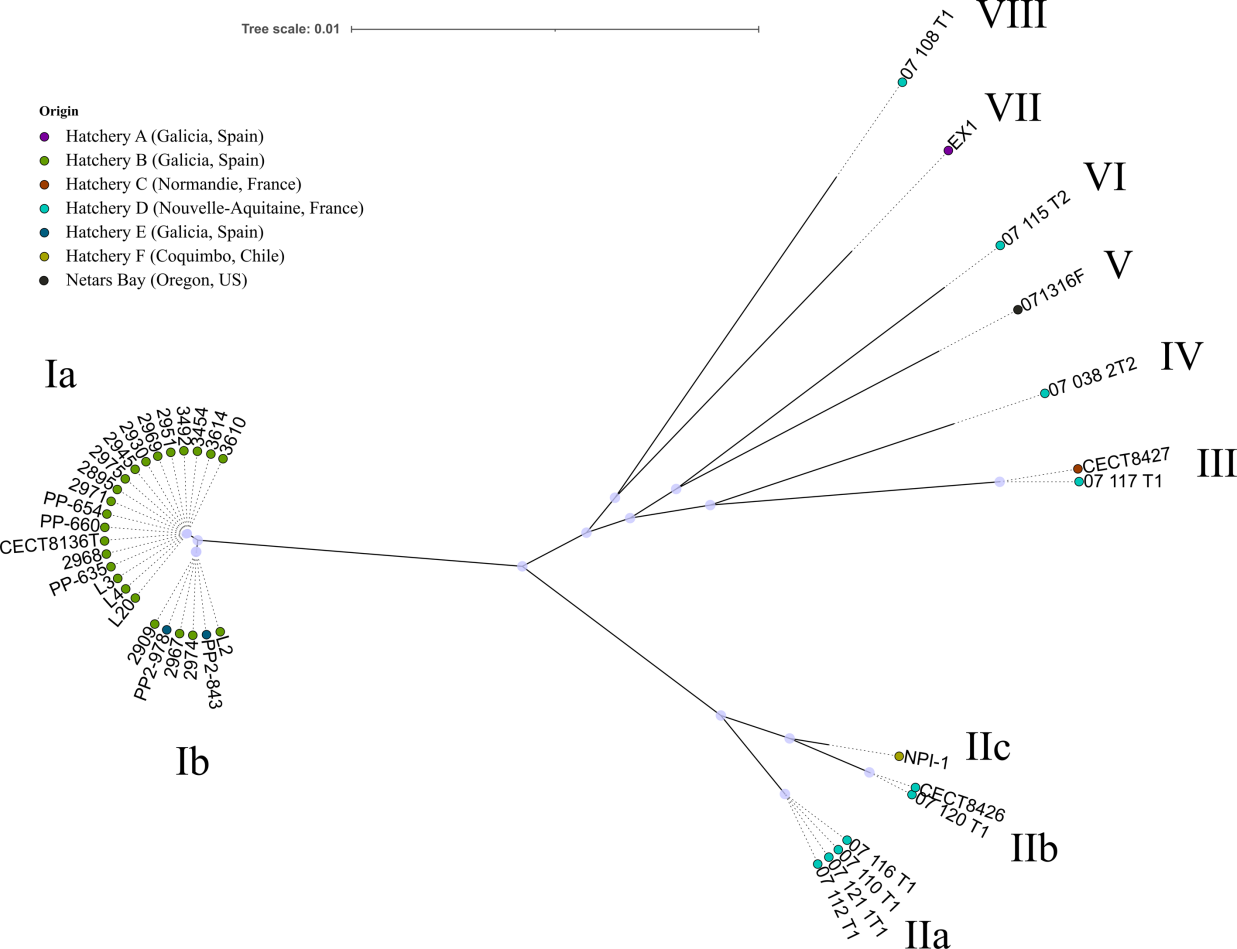
Phylogenomic tree of *V. europaeus*. The tree was built by the concatenation of 3,846 concatenated core genes. Nodes with significant bootstrap values (*n*=100) are marked with a light purple dot. Clusters are indicated with Roman numerals (I–VIII).

The phylogenomic tree based on the concatenated sequences of the 3,846 core genes showed a topology with three main branches containing a total of eight robust clusters (bootstrap value=100; terminal or subterminal branches distances >0.006): branch I (cluster I), branch II (cluster II) and branch III (clusters III–VIII), with clusters I and II divided into subclusters (bootstrap value=100; terminal branch distances from 0.0003 to 0.0025) (Fig. 2). These phylogenetic relationships were supported by ANIb analysis; ANI values were higher than 0.979 in all pairwise comparisons and around 0.995 for the strains belonging to the same cluster including between closely related subclusters (Fig. S3). Interestingly, analyses of the phylogeny based on accessory genes showed the same eight clusters, although, as expected, the diversity was higher than that of the core genome phylogeny (Fig. S4).

**Fig. 3.**
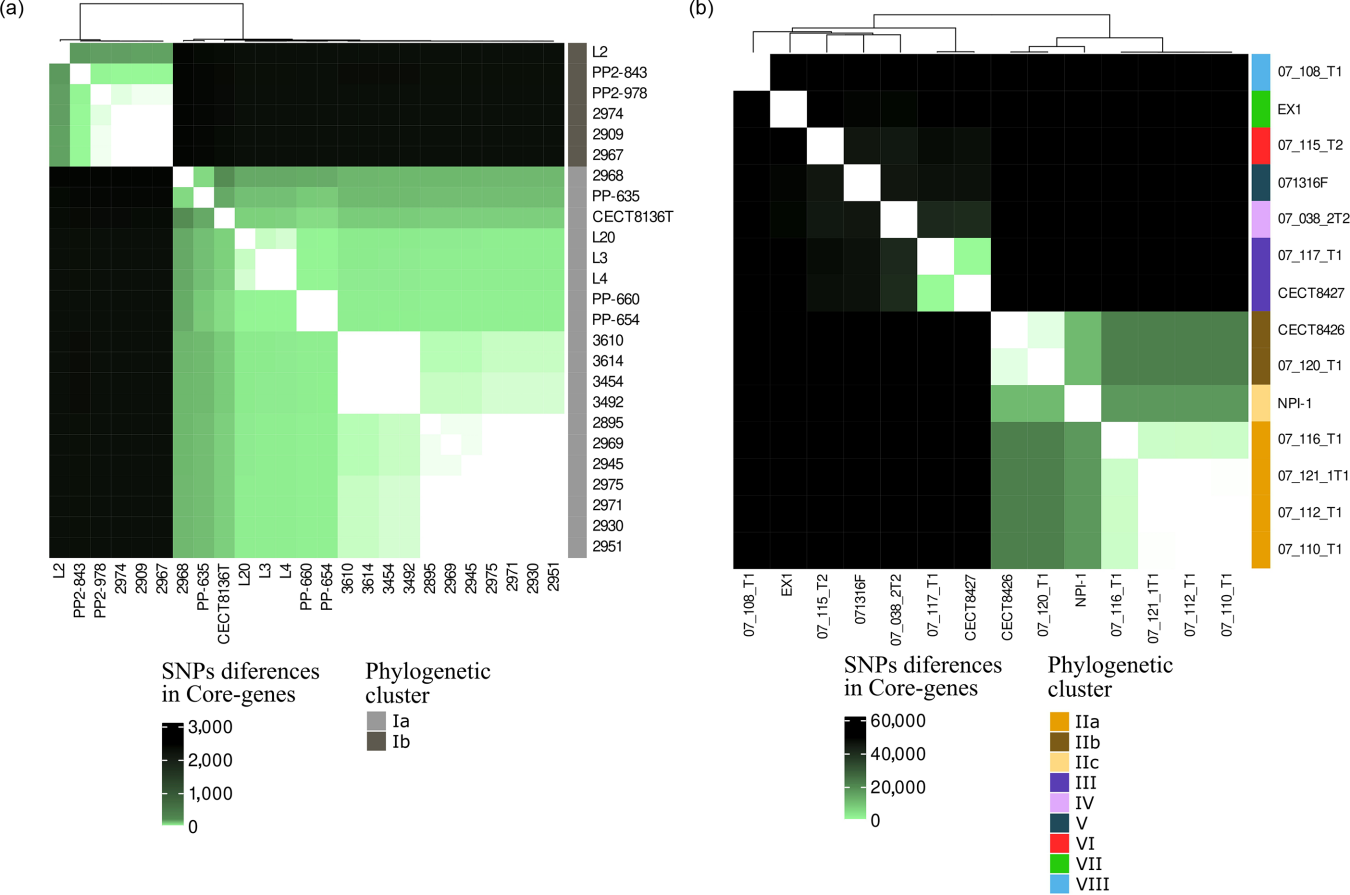
Heatmaps showing the genetic variability based on the SNP differences in the core genes of the *V. europaeus* strains belonging to (**a**) Subclusters Ia and Ib and (**b**) clusters from II to VIII. In both heatmaps, phylogenetic clusters and subclusters are shown in the right bar.

Cluster I was the largest group (25 strains), and it was exclusively constituted by the strains isolated from the Spanish hatcheries B (23 strains) and E (2 strains) (Table 1). Those strains were distributed into two subclusters: Ia (19 strains) and Ib (6 strains). The cluster comprises strains isolated in different years (2001, 2008, 2011 and 2018), from a broad host range and stages of the life cycle of bivalves such as eggs, larvae, spat (or juveniles) and adults (Fig. 2, Table 1). A complementary analysis to assess genetic differentiation and diversity within and between clusters was performed using SNPs detected in the coding regions of the core genome. Nucleotide diversity between subclusters Ia and Ib was low, including a maximum of 197 SNPs and 162 SNPs among the strains belonging to subcluster Ia (CECT8136T vs. 2968) and subcluster Ib (2974 vs. L2), respectively; however, more than 2,300 SNPs were identified between both subclusters (Fig. 3a). Furthermore, analyses of the core-genome SNP dataset revealed the existence of many clonal Spanish strains (Table 2), isolated from: (i) the same bivalve species (but from different samplings) within subcluster Ia and subcluster Ib, (ii) broodstock to the offspring after spawning (vertical transmission) and (iii) different hatcheries, hosts and/or dates. In contrast, there were SNP differences among strains isolated from the same sampling, despite their assignment to the same subcluster, such as 89 SNPs between PP-635 and PP-660/PP-654, 146 SNPs between PP-635 and CECT8136 or 118 SNPs between 2968 and 2895/2930/2945/2951/2969/2971/2975 (Fig. 3a).

**Table 2.** Clonal strains identified from SNP analysis of the *V. europaeus* core genome

Isolated from	Location:	Host	Cluster	Clonal strain	SNP difference
Same bivalve species	Hatchery B (Spain)	*D. trunculus* (larvae and seawater)	Ia	3454, 3492	0
	Hatchery B (Spain)	*R. decussatus* (larvae and seawater)	Ia	2895, 2930, 2945, 2951, 2969, 2971, 2975	1–2
	Hatchery B (Spain)	*E. arcuatus* (larvae)	Ia	L3, L4	5
	Hatchery B (Spain)	*O. edulis* (larvae)	Ia	PP-654, PP-660	1
	Hatchery B (Spain)	*R. decussatus* (larvae)	Ib	2909, 2974, 2967	1
	Hatchery E (Spain)	*R. philippinarum* (spat)	Ib	PP2-843, PP2-978	14
	Hatchery D (France)	*M. gigas* (spat)	IIa	07/110 T1, 07/112 T1, 07/121 T1	2
	Hatchery D (France)	*M. gigas* (spat)	IIb	07/120 T1, CECT8426	28
Vertical transmission	Hatchery B (Spain)	*R. decussatus* broodstock to eggs	Ia	3610, 3614	0
Different hatcheries/hosts/dates	Hatchery E and B (Spain)	*R. philippinarum* vs. *R. decussatus*	Ib	PP2-978 vs. 2909/2974/2967	3
	Hatchery B (Spain)	*R. philippinarum* vs. *E. arcuatus*	Ia	L20 vs. L3/L4	5
	Hatchery B (Spain)	*D. trunculus* vs. *R. decussatus*	Ia	3454/3492 vs. 3610/3614	1
	Hatchery B (Spain)	*R. decussatus* vs. *D. trunculus*	Ia	2895/2930/2945/2951/2969/2971/2975 vs. 3454/3492	5
	Hatchery B (Spain)	*R. decussatus* (larvae) vs. *R. decussatus* (broodstock and eggs)	Ia	2895/2930/2945/2951/2969/2971/2975 vs. 3610/3614	6
	Hatchery B (Spain)	*O. edulis* vs. *R. philippinarum* vs. *E. arcuatus*	Ia	PP-654/PP-660 vs. L20; PP-654/PP-660 vs. L3/L4	19

EX1 constituted the first known isolation of *V. europaeus* (year=1985) and the only isolate from the Spanish Hatchery A (Table 1). This could support that EX1 was the only Spanish strain located outside cluster I, and that it constituted a well-differentiated group (cluster VII) in branch III, closer to the French strains than to the Spanish strains (Fig. 2).

Conversely, most of the French strains (10/11 strains) were isolated from the same geographical origin and similar time (Table 1); however, they exhibited much more genetic diversity than the Spanish strains (Fig. 2). Those ten French strains were distributed among six different clusters: branch II (cluster II=subclusters IIa, IIb and IIc) and branch III (clusters III, IV, VI and VIII) (Fig. 2). SNP analyses also revealed the existence of clonal French strains within the clusters IIa and IIb (Table 2). Cluster III (Fig. 2) was formed by two strains (07/117 T1 and CECT8427) from two different origins (Hatchery D and Hatchery C, located in Nouvelle-Aquitaine and Normandy, respectively, Table 1) and hosts (*Magallana gigas* spat and the non-bivalve species *Haliotis tuberculata,* Table 1) identifying 182 SNPs among them (Fig. 3b). Other strains isolated from Hatchery D (07/138 2T2, 07/115 T2 and 07/108 T1) constituted independent clusters by themselves (clusters IV, VI and VIII) supported by differences of more than 50,000 SNPs with their contemporary strains belonging to subclusters IIa and Iib and cluster III (Fig. 3b).

Despite the Chilean strain NPI-1 being closely related to subcluster IIb, it constituted the subcluster IIc (Fig. 2) supported by a difference of 11,208 SNPs with its closest relatives (Fig. 3b). The American strain 071316F was the only one not isolated from a hatchery environment, and it also grouped in an independent cluster (cluster V), sharing a node with the French strain 07/115 T2 (Fig. 2).

### The key virulence factors are encoded in the core genome

**Fig. 4.**
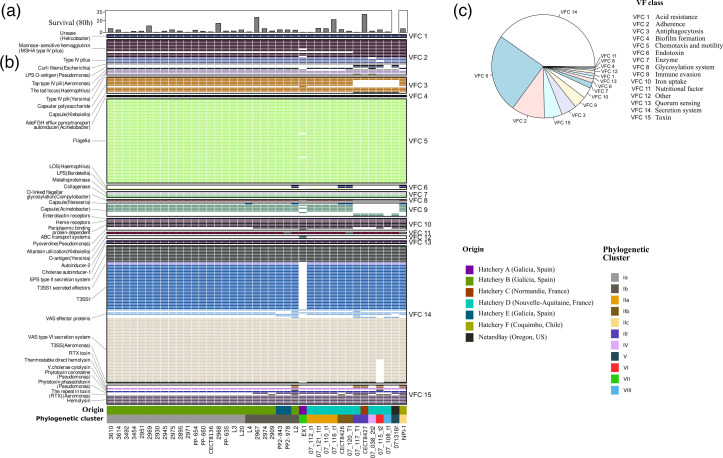
Virulence genes identified from the *V. europaeus* genomes: (**a**) survival of Manila clam juveniles (%) after *V. europaeus* infection. (**b**) Presence/absence of virulence genes. (**c**) Relative abundance of the different virulence factor classes (VFCs) in the *V. europaeus* pangenome. Virulence factors are indicated on the left and VFCs on the right. The geographical origin of each *V. europaeus* strain and its phylogenetic clusters is indicated at the bottom.

According to the virulence challenges, all the *V. europaeus* strains tested (38 strains) were virulent against Manila clam juveniles (Fig. 4a). Survival rates were low in all cases, ranging from 0 to 7% in most cases (32/38 strains), 22 to 27% for 3/38 strains (CECT8427, 07/116 T1 and 2967) and 9 to 16% for 3/38 strains (07/121 1T1; 2968 and 2969) (Fig. 4a).

A total of 231 genes (Fig. 4b; Table S4) were identified as potential virulence factors from the *V. europaeus* pangenome, and those were distributed in 15 virulence factor classes (VFC 1–15) (Fig. 4b and c). Among these genes, 59.3% (137 genes) were assigned to the core genome. Core virulence genes (Fig. 4b, Table S4) were related to acid resistance (VFC 1), adherence (VFC 2), antiphagocytosis (VFC 3), biofilm formation (VFC 4), chemotaxis and motility (VFC 5), enzymes (VFC 7), iron uptake (VFC 10), quorum sensing (VFC 13), secretion systems (VFC 14) and toxins (VFC 15). In contrast, other VFCs, such as endotoxins (VFC 6), glycosylation system (VFC 8), immune evasion (VFC 9), nutritional factors (VFC 11) and others (VFC 12), did not contain any gene assigned to the core genome.

It is important to remark that three classes, VFC 14 (secretion systems), VFC 5 (chemotaxis and motility) and VFC 2 (adherence), comprised 75% of the virulence factors (Fig. 4b and c). Particularly, the pangenome included the whole gene machinery of five secretion systems (Fig. 4b, Table S4): T2SS, T3SS and three different T6SS (T6SS1, T6SS2 and T6SS3). From those, the T2SS, T6SS1 and T6SS3 belonged to the core genome (Fig. 4b). T6SS2 and T3SS were assigned to the accessory genome, despite being encoded by most *V. europaeus* strains, only the French strain 07/115 T2 was defective for T6SS2 and the Spanish strain EX1 was defective for T3SS (Fig. 4b). Despite both T6SS1 and T6SS2 showing a similar synteny and belonging to the T6SSi5 subtype, they presented significant differences among gene homologues (Fig. S5). T6SS3 was different from T6SS1 and T6SS2; it was classified into the T6SSi1 subtype (Fig. S5).

**Fig. 5.**
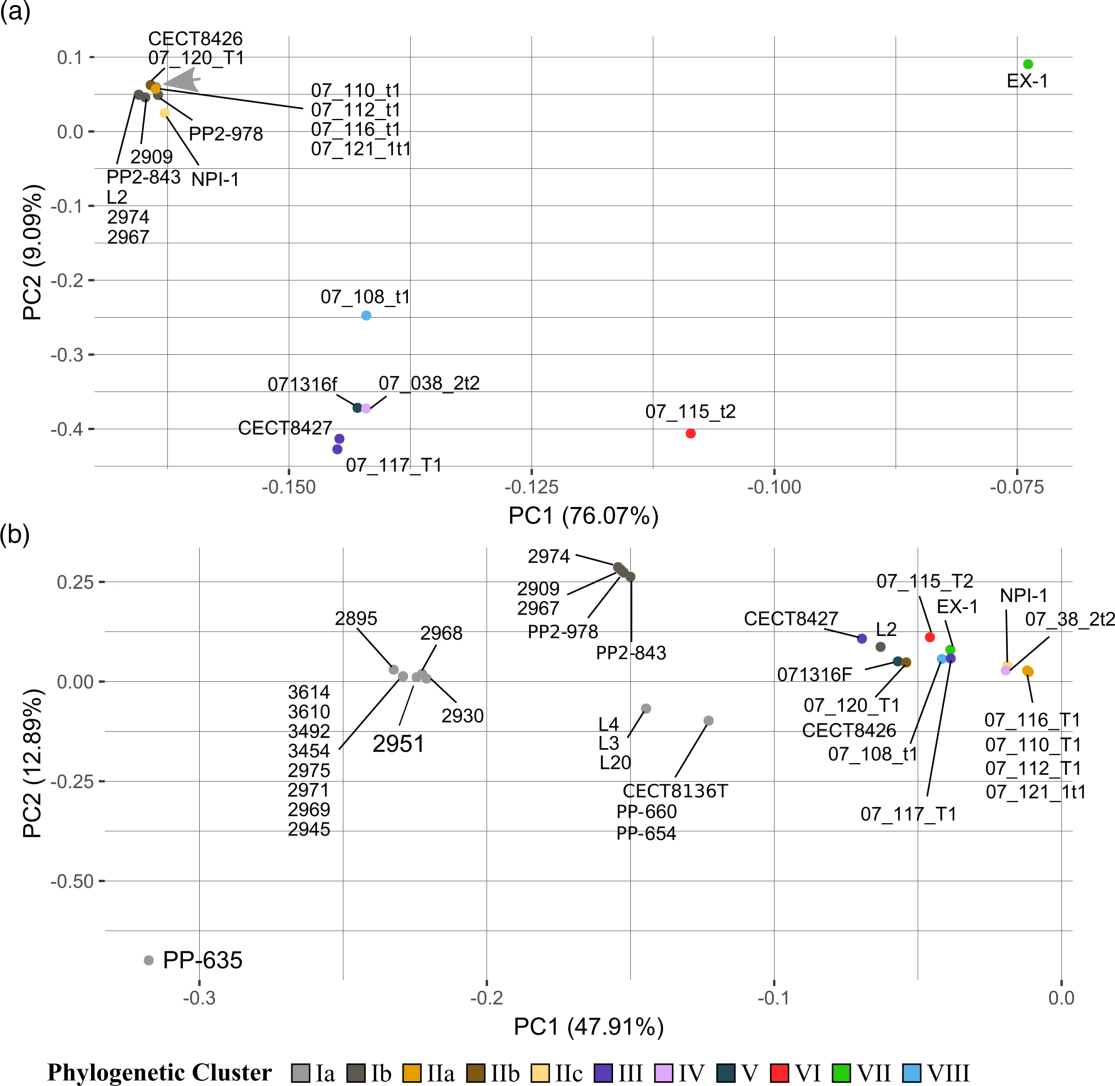
Genetic variability of *V. europaeus* strains based on the virulence factors (**a**) and anti-phage defence systems (**b**) encoded in the accessory genome. Phylogenetic clusters based on the core genome were shown in different colours in PCA. The grey arrow in (**a**) indicates the position in the PCA of the strains: PP-660, PP-635, CECT8136, PP-654, 2895, 2945, 2968, 2930, 2951, 2969, 2971, 2975, 3610, 3492, 3454, 3614, L3, L4 and L20.

**Fig. 6.**
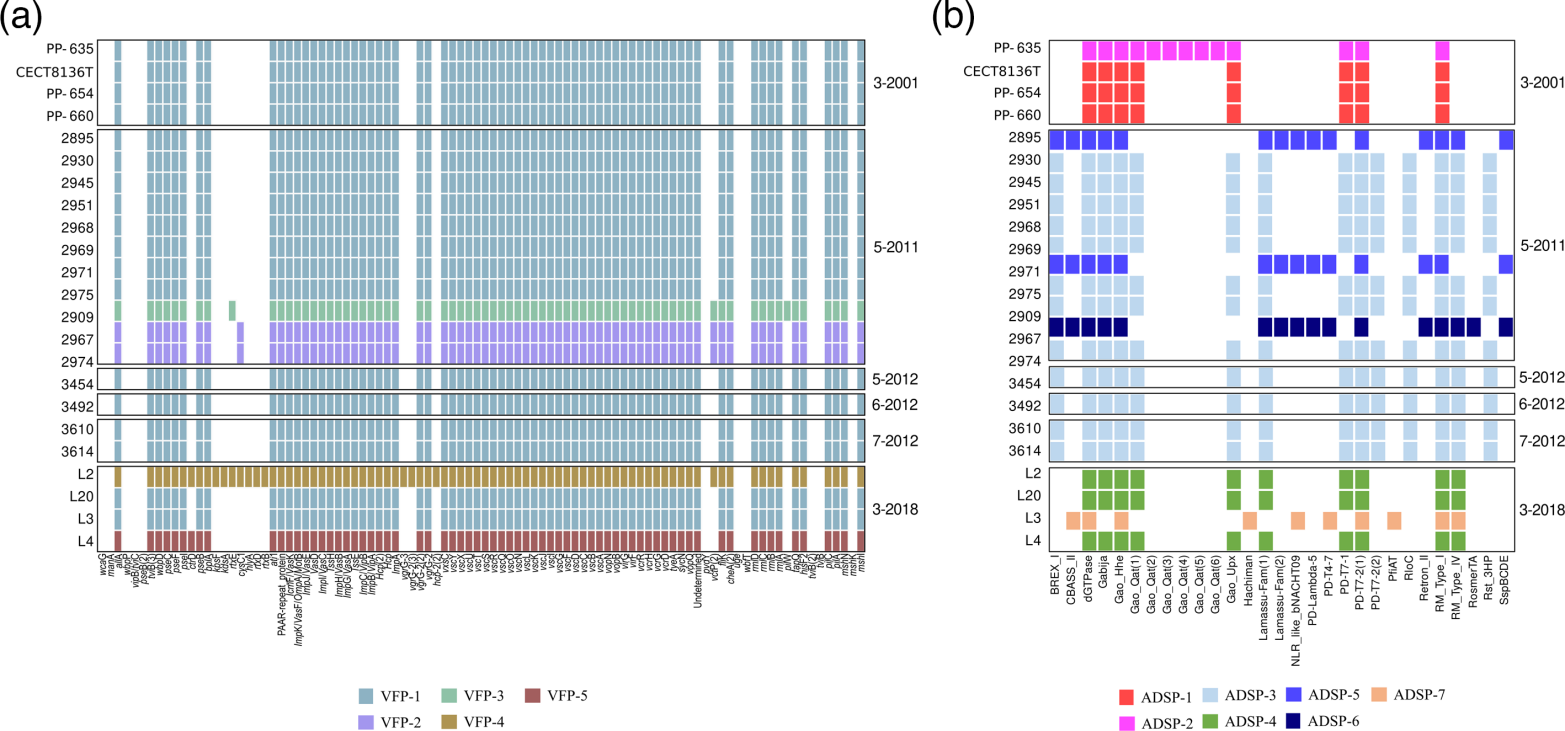
Co-existence of different virulence factor profiles (**a**) and anti-phage defence systems (**b**) in Hatchery B (Spain) along the time series (2001–2018). The presence/absence of accessory virulence-related genes was displayed in rows, and the different profiles (VFP 1–5 or ADSP 1–7) were indicated by colours. (**a**) Virulence factor profiles (VFP 1–5), VFP 2 and VFP 3 were very similar to VFP1; however, they differed in the presence of VAS effector protein coding genes *vgrG-2*, the allantoin utilization gene *allA* and the presence/absence of some toxins such as CysC1 (present only in VFP 3) or RtxE (present only in VFP 2). VFP5 was identical to VFP 1, but it encoded an additional non-core immune evasion gene, *ctrD*. VFP 4 included 12 additional non-core virulence genes in comparison with VFP 1, such as those encoding an additional VAS effector protein of the T6SS encoded in the accessory genome (*hcp-2_2*, *vgrG-2*, *vgrG-2_3* and *vgrG-3*), toxins (*rtxB*, *rtxD*, *rtxE*, *hlyA* and *cysC1*), endotoxins (*kdsA* and *kpsF*), immune evasion (*ctrD*) and allantoin utilization (*allA*). (**b**) Anti-phage defence systems profiles (ADSP 1–7) included the following systems: ADSP 1 (dGTPase, Gabija, Gao Hhe, Gao Qat, Gao Upx, PD T7 1, PD T2 7 and RM type I); ADSP 2 was similar to ADSP1 but ADSP2 harboured five additional copies of the Gao Qat system; ADSP 3 was similar to ADSP 1, encoding additional defence systems such as BREX I, Lamassu Fam, RloC, RM type IV and Rst_3HP; ADSP 4 was similar to ADSP 1, encoding also Lamassu Fam and RM type IV; ADSP 5 and ADSP 6 shared 14 defence systems, BREX I, CBASS II, dGTPase, Gabija, Gao Hhe, Lamassu Fam (two copies), NLR-like bNACHT09, PD lambda 5, PD T7 2, Retron II, RM type I, RM type IV and SspBCDE; but ADSP5 (2909 and 2967) also encoded PD T4 7. ADSP6 (2974) encoded additional systems such as PD T4 7 and RosmerTA; ADSP7 (L2) showed a very different profile than its contemporary strains L3, L4 and L20, encoding a total of eight defence systems (CBASS II, dGTPase, Gao Hhe, Hachiman, NLR-like bNACHT09, PD T4 7, PfiAT and RM type I), and it was the only one encoding the PfiAT system.

The PCA analysis (Fig. 5a) explained 85.16% of the genetic diversity of the non-core virulence factors. Thereby, genetic variability based on the non-core virulence factors was low, and they showed differences only in the presence/absence of a few virulence genes among closest relatives (Table S5). The co-existence of different virulence profiles in the same environment could be evaluated in the Hatchery B; in this case, the strains obtained in this facility, isolated from different cultures along the time-series, presented different profiles (2001–2018) (Fig. 5a). Five different virulence factor profiles were identified in this Hatchery (VFP 1–5), although they were very similar, differing mostly in single genes (Fig. 5a). For instance, VFP 1 was always detected along the time-series, being the only profile in Mar/2001, May/2012, Jun/2012 and Jul/2012, and those strains shared 70 non-core virulence factor genes. VFP 1 co-existed with VPF 3 and VFP 4 in May/2011 and with VFP 2 and VFP 5 in Mar/2018 (Fig. 6a). Interestingly, the strain L2 represented VFP4, and it harboured the highest number of virulence genes (82 genes) among the *V. europaeu*s strains (Fig. 6a).

French strains showed a higher variability based on non-core virulence genes than the Spanish strains, but lower than that observed in the core genome phylogeny. For instance, strains belonging to subcluster IIa (07/112 T1, 07/121 1T1, 07/110 T1 and 07/116 T1) were close to Spanish strains (subclusters Ia and Ib) (Fig. 5a). Interestingly, strains 07/115 T2 (cluster VI) and EX-1 (cluster VII) showed the most different virulence profiles (Fig. 5a) because they encoded a lower number of non-core virulence genes (07/115 T2=57 genes; EX1=38 genes) than their closest relatives, since they were defective for T6SS2 and T3SS, respectively.

### Antimicrobials are (still) effective to fight *V. europaeus*

A consensus between the *in silico* and phenotypic results was established to determine the antibiotic resistance profile. All *V. europaeus* strains were sensitive to most of the antimicrobials evaluated in this study, including antimicrobials assigned to the antibiotic classes rifamycin, cephalosporin, beta-lactam, polymyxin, quinolone, amphenicol, streptogramin A, folate pathway antagonist, pleuromutilin, glycopeptide, steroid antibacterial, macrolide, streptogramin B, oxazolidinone, aminoglycoside, fosfomycin, nitroimidazole, aminocyclitol, pseudomonic acid, tetracycline and lincosamide (Table S6). In contrast, all *V. europaeus* strains available in our lab collection (38 strains) were resistant to erythromycin according to the disc diffusion method, which was confirmed with the presence of the antibiotic resistance gene (ARG) *crp* (Table S6). Interestingly, some strains showed additional resistance phenotypes, such as the isolates 3454, 3492, 3610, and 3614, which were also resistant to streptomycin (S, 10 µg) and sulfonamide (SULDD, 25 µg), bestowed by the presence of the ARGs *aph(3″)-Ib* and *aph(6)-Id* genes and *sul2* gene, respectively, in their genomes (Table S6).

Some discrepancies were found between *in silico* and phenotypic results, and thus *in silico* data were curated with the results obtained by the disc-diffusion method (Table S6). For example, despite no cephalosporin resistance gene being found in the CARD and ResFinder databases, all *V. europaeus* strains tested were experimentally resistant to cephalexin on MHA-1 plates. On the other hand, some resistance genes found by *in silico* analyses rendered a negative phenotypic result: (i) the *floR* gene was identified in the genomes of the strains 3454, 3492, 3610 and 3614 by ResFinder (identities ranged from 98.19 to 98.27%), however, they were sensitive to florfenicol (FFC, 30 µg); (ii) tetracycline resistance genes were also found in all strains from both databases (gene identity >85%); however, they were sensitive to tetracycline (TE, 30 µg).

### *V. europaeus* produces an important number of secondary metabolites, mostly encoded in the accessory genome

**Fig. 7.**
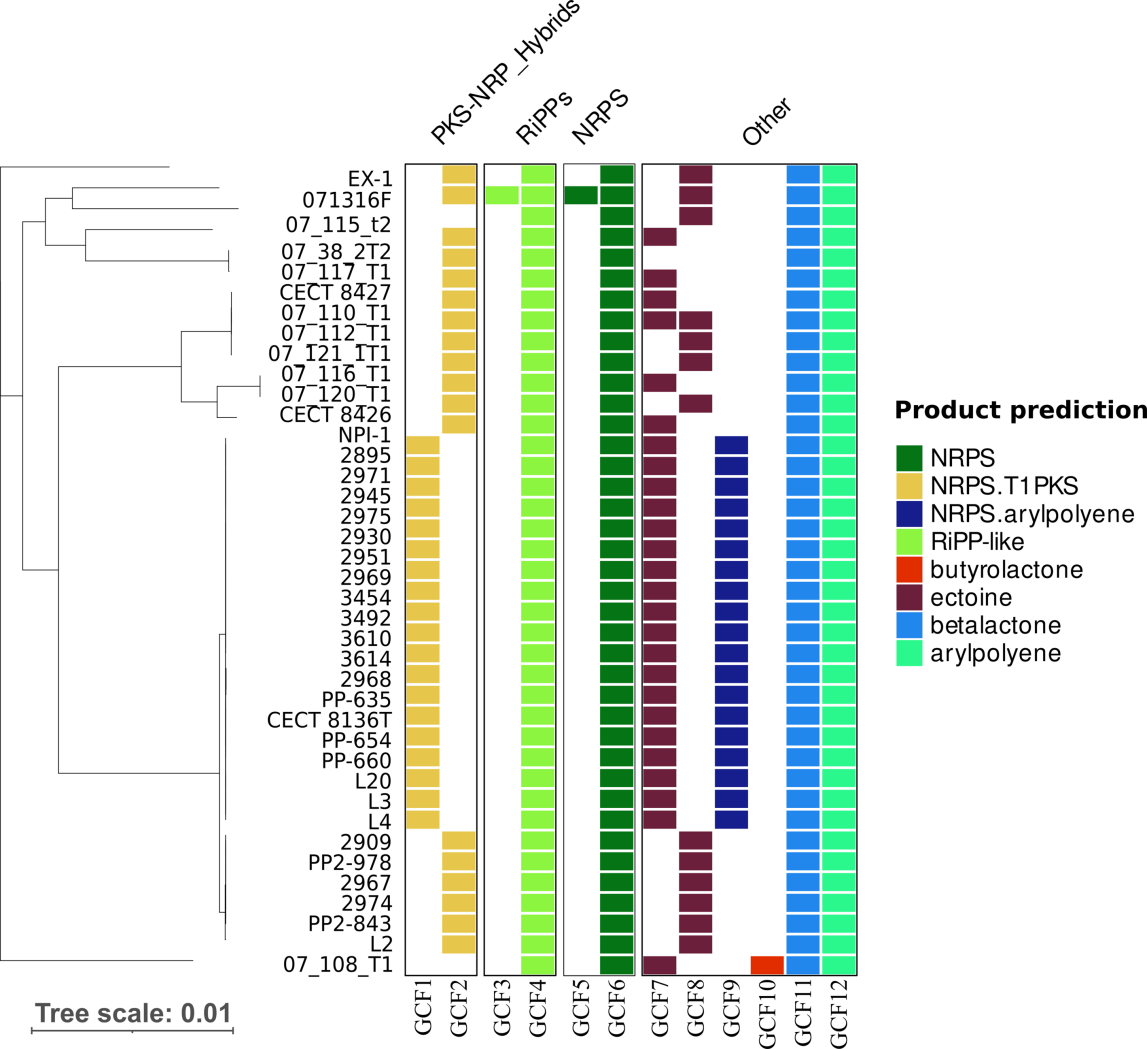
Heatmap showing the biosynthetic GCFs encoded by each *V. europaeus* strain. Products from each GCF are indicated by different colours. The core genome phylogeny dendrogram indicating the clustering of the different GCF profiles is shown on the left.

*V. europaeus* genomes encoded a total of 254 BGCs. Most of the strains encoded 6–7 BGCs, ranging from the five BGCs encoded by the French strain 07/115 T2 (cluster VI) to the eight BGCs of the American strain 071316F (cluster V) (Fig. 7). The study of the intra-BGC diversity allowed its classification into four major classes encoding the following BGCs (%): PKS-NRP hybrids (14.57%), RiPPs (15.75%), NRPS (15.75%) and others (53.94%). Within those classes, 12 biosynthetic GCFs could be defined (Fig. 7): GCF1 and GCF2 presumably produced PKS-NRP hybrids; GCF3 and GCF4, post-translationally modified peptides (RiPPs); GCF5 and GCF6, non-ribosomal peptide synthetases (NRPS); GCF7 and GCF8, ectoine; GCF9, arylpolyene-NRPS hybrids; GCF10, butyrolactone; GCF11, betalactone; and GCF12, arylpolyene. Diversity among BGCs within the same GCF is shown in Fig. S6

GCF4, GCF6, GCF11 and GCF12 contained BGCs encoded by all *V. europaeus* strains, and, thus, they were assigned to the core genome (Fig. 7). GCF1, GCF2, GCF7, GCF8 and GCF9 were conformed by sequences of just a subset of strains (Fig. 7): GCF1 and GCF9, encoded only by the Spanish strains belonging to subcluster Ia; GCF2, encoded by Spanish, French, Chilean and American strains (clusters Ib, IIa-c, III, IV, V and VII); GCF7, encoded by Spanish, French and Chilean strains (clusters Ia, IIa-c, III, VIII and IV); and GCF8, encoded by Spanish, French and American strains (clusters Ib, IIa-b, V, VI and VII). Three GCFs were encoded by a single genome: GCF3 and GCF5 were only encoded by the American strain 071316 f (US; phylogenetic cluster V), whereas GCF10 was only detected in the French strain 07/108 T1. According to antiSMASH results, GCF5 presented a 100% similarity to the siderophore amphibactin B biosynthetic gene cluster of *Vibrio neptunius*, and GCF12 presented an 85–90% similarity to the arylpolyene Vf BGC of *Aliivibrio fischeri* ES114.

### Variability of *V. europaeus* is explained by the presence of anti-phage defence systems in the accessory genome

**Fig. 8.**
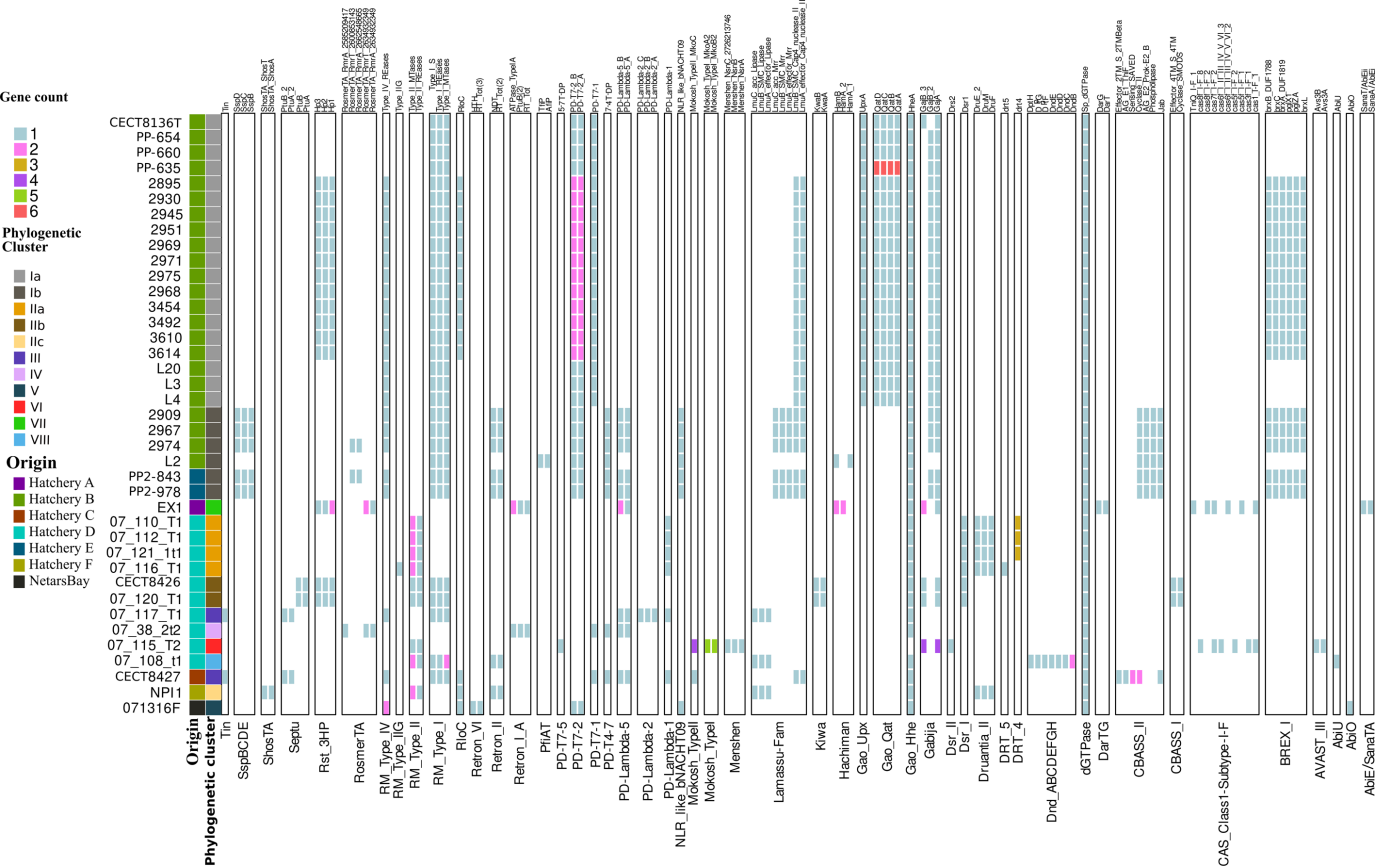
Anti-phage defence systems encoded by *V. europaeus* strains. Components are coloured by the number of copies and grouped by a specific defence system. The geographical origin of the *V. europaeus* strains, the phylogenetic cluster based on the core genomes and the anti-phage defence system profiles are indicated on the left.

*V. europaeus* genomes were enriched in anti-phage defence systems: a total of 49 systems were identified among the studied genomes (Fig. 8). It is important to remark that anti-phage defence systems were mostly encoded by the accessory genome, and only the dGTPase system was identified in the core. Interestingly, the Gao Hhe system was absent only in the French strain 07/115 T2 (Fig. 8).

PCA analyses (Fig. 5b) revealed that the genetic variability among *V. europaeus* strains is mainly determined by the anti-phage defence systems, in comparison with other accessory genes, such as virulence genes, ARG or BGCs. For instance, different anti-phage defence system profiles (ADSPs) co-existed to effectively protect the *V. europaeus* population from phage predation in Hatchery B, and ADSP variability along the time series was higher than that observed for the virulence profiles (five different VFP types vs. seven different ADSP types) (Fig. 6a and b). However, it is important to remark that anti-defence systems are formed by several genes in most cases. In Hatchery B, ADSP 1 and ADSP 2 profiles co-existed in March 2001 (Fig. 6b). Defence systems encoded by ADSP 1 were common in other new profiles such as ADSP 3 and ADSP 4 (Fig. 6b). ADSP 3 was the only profile detected in strains isolated in May, June and July 2012, and it co-existed with ADSP 5 and 6 in May 2011 (Fig. 6b). Interestingly, ADSP5 (strains 2909 and 2967) was the most protected phenotype since it included the highest number of anti-phage defence systems, with a total of 16 full systems. Besides, ADSP 7 (strain L2) co-existed with ADSP 4 and showed a very different profile than its contemporary strains L3, L4 and L20 (Fig. 6b).

French strains isolated from Hatchery D also showed high variability, supporting the co-existence of different profiles in the same hatchery (Fig. 5b). Within the subcluster IIa, three strains 07/110 T1, 07/112 T1 and 07/121 1T1 encoded seven systems such as dGTPase and three copies of DRT 4, Druantia II, Dsr I, Gao Hhe, PD lambda I and RM type II. The remaining strain belonging to this subcluster (strain 07/116 T1) showed a similar profile; however, it harboured two additional systems, being the only *V. europaeus* strain encoding the DRT 5 and RM type IIG systems (Fig. 8). Strains belonging to subcluster IIb (strains CECT8426 and 07/120 T1) encoded ten systems (CBASS I, dGTPase, Dsr I, Gabija, Gao Hhe, Kiwa, RM type I, RM type II, Rst 3HP and Septu). The remaining strains isolated from Hatchery D (Fig. 8), such as 07/117 T1 (cluster III), 07/038 2T2 (cluster IV), 07/115 T2 (cluster VI) and 07/108 T1 (cluster VIII) encoded exclusive defence systems, for example, (i) PD-Lambda-2 system only encoded by 07/117 T1; (ii) Retron IA system only encoded by 07/038 2T2, which was the strain with the lowest number of defence systems, with only six full systems; (iii) AVAST III, Dsr II, Menshen and Mokosh_Type I only encoded by 07/115 T2; and (iv) AbiU and Dnd ABCDEFGH systems only encoded by 07/108 T1.

The remaining *V. europaeus* strains encoded exclusively specific defence systems. For instance, the Spanish strain EX1 (cluster VII), isolated from Hatchery A, was the only one encoding the DarTG and SanaTA systems (Fig. 8), whereas the only French strain isolated from a different hatchery (strains CECT8427; Hatchery C, Normandy, Fig. 8) encoded 11 defence systems (CBASS II, dGTPase, Gao Hhe, Lamassu Fam, PD-lambda 1, PD-lambda 5, PD-T7 1, RloC, RM_Type_I, RM Type IV and Septu), and it was the only one with two copies of the genes of CBASS (Fig. 8). The American strains NPI-1 (Chile; cluster IIc) and 071316F (USA; cluster V) encoded a total of seven and nine defence systems, respectively. Interestingly, some defence systems were only harboured by those strains, such as AbiO and Retron VI systems only encoded by the strain 071316F or the ShosTA system, encoded only by the Chilean strain NPI-1.

## Discussion

The pangenome analyses presented here are based on a dataset that includes genomes with varying assembly levels, ranging from complete genomes to draft assemblies. Importantly, all assemblies used in this study were highly confident and suitable for pangenome analyses, as genome completeness and contamination fit the quality thresholds proposed by Parks *et al.* [22] (≥95% completeness and ≤5% contamination). Variation in genome fragmentation may influence gene prediction and clustering, potentially leading to gene splitting or underestimation of accessory gene content in more fragmented assemblies.

The pangenome obtained for different species of the genus *Vibrio* showed a broad size range [20,44,47], reflecting a high genomic diversity within the genus, which matches its well-known ecological diversity [48,49]. The pangenome of *V. europaeus* (9,860 genes) was comparable in size to those reported for other *Vibrio* species analysed using a similar number of genomes, such as the multi-host pathogen *Vibrio anguillarum* (9,537 genes across 44 genomes) [46]. In contrast, other *Vibrio* species, such as *Vibrio tapetis*, exhibited larger pangenomes (11,213 genes), despite being analysed using a smaller dataset (*n*=17 genomes) [20]. Regarding the percentage of core genes in the pangenome, it is strongly related to the evolutionary history and lifestyle of a bacterial species. For instance, free-living species with a high degree of dispersion have a small proportion of core genomes [1]. In our study, *V. europaeus* exhibited a higher core genome (39.00%) than other *Vibrio* pathogens with a broad host range, such as *V. anguillarum* (28.24%), *V. tapetis* (29.90%) or *Vibrio fluvialis* (15.36%) [20,46, 47]. This can be justified due to the bias of the *V. europaeus* collection because most of the strains were isolated (i) from marine mollusks but not from other phyla, (ii) associated with massive mortalities but not from healthy animals and (iii) from a reduced number of locations. The inclusion of closely related or clonal strains may bias estimates of core genome size and pangenome openness by inflating shared gene content. For these reasons, pangenome metrics are interpreted in the context of the observed population structure and should be regarded as descriptive of gene content diversity within the currently available genomic dataset of *V. europaeus*, rather than as definitive estimates of the species-wide pangenome.

Analysis of the core genome allowed us to evaluate the evolutionary history and intraspecific diversity of *V. europaeus*. Specifically, the Spanish strains belonging to phylogenetic subclusters Ia and Ib displayed a lower genetic variability than the French strains. As observed previously by Campbell [50] for the radiation event of the clonal type of *Vibrio parahaemolyticus*, those Spanish populations spread from a monophyletic radiation event that was recently dispersed artificially or naturally throughout the hatcheries under study (Hatchery B and E). In contrast, French strains exhibited a higher variability than the Spanish strains, even though the geographical and temporal distribution of their isolation was more restricted in time and space (Hatchery D and samplings performed in August 2007). In the case of Chilean strain NPI-1, its closeness to the French strains of subcluster IIb strongly suggests an intercontinental transfer of *V. europaeus*, probably mediated by anthropic movement (molluscs’ spats, broodstock and phytoplankton). Likewise, strain EX1 reflects historical practices in hatchery management; during that period, broodstock were mostly imported from France, facilitating the introduction of genetically diverse *V. europaeus* lineages (personal communication, Juan L. Barja). This explains the distinct placement of strain EX1, clustering closer to French isolates rather than Spanish strains. This anthropic movement event would also support the finding of clonal strains in different locations in Spain and France.

Comparative analyses revealed that 60% of the virulence genes of the *V. europaeus* pangenome belonged to the core genome. It is important to remark that all *V. europaeus* strains were demonstrated to be highly virulent towards Manila clam juveniles; consequently, this led to the determination that the key virulence factors are necessarily encoded by the core genome. In the case of *Vibrio* pathogenic to bivalves, pathogenesis is multifactorial and depends on the combination of different virulence factors, such as successful chemotaxis, adherence and initial colonization of bivalve tissues; survival against the bivalve immune system and proliferation in haemolymph; and colonization of the connective tissue, bacterial proliferation and nutrient acquisition, which finally causes tissue disruption and host death [10,12]. It is important to note that the virulence factors of *V. europaeus* were strictly analysed here by comparative genomics. However, genetic manipulation will be essential to demonstrate the functional role of those genes in virulence. Due to their presence in all *V. europaeus* strains, key pathogenic effectors seem to be driven by the core secretion systems T2SS and both T6SS (T6SS1 and T6SS3). Virulence-related functions of T2SS include the participation in attachment, biofilm formation and colonization, as well as the secretion into the extracellular medium of proteins capable of producing tissue disruption, including proteases, pectinases, phospholipases, lipases and toxins [51,52]. The T6SS plays a major role in interbacterial competition and in bacterial interactions with eukaryotic cells [53,54].

Interestingly, 97% of the *V. europaeus* strains encoded three different T6SSs (T6SS1–3), which is an unusual phenomenon. T6SS1 and T6SS3, belonging to the core genome and encoded by chromosome 1, appeared to be widespread among all the strains. In contrast, T6SS2 was assigned to the accessory genome, and, thus, it was probably acquired later by a horizontal gene transfer event or resulted from a specific gene loss within that phylogenetic group [55,57]. T6SS2 and T6SS3 are similar to the previously reported T6SS3-like of *Vibrio proteolyticus* and *V. parahaemolyticus* involved in delivering anti-eukaryotic effector proteins into mammalian phagocytic cells [55,58]. Consistent with this, Martínez [59] recently identified T6SS3-associated proteins in the secretome of *V. europaeus*, and experimental infections using the total secretome in Manila clam juveniles resulted in mortality rates approaching 80%, suggesting a key role for this core system in virulence. T6SS1 is similar to the T6SS1-like of *Vibrio coralliilyticus*, which mediates antibacterial activities [60,61]. Our findings revealed a diverse repertoire of T6SSs in the *V. europaeus* pangenome, which can play a major role in the interactions of this species with other cells.

On the other hand, the T3SS, a recognized virulence factor of Gram-negative bacteria capable of injecting effectors of variable functions into host cells [62,64], is present in all the *V. europaeus* strains except for the oldest isolate (strain EX1), suggesting a recent horizontal acquisition of that operon [65]. The T3SS identified in *V. europaeus* corresponded to the T3SS1 described in *V. parahaemolyticus*; this has been reported to be mainly related to biofilm formation, motility and cytotoxicity [66,67]. Due to the remarkable absence of T3SS in the pathogenic strain *V. europaeus* EX1, its role in the pathogenicity of the species deserves further investigation.

In relation to other virulence factors, *vemA* and *prtV* genes (encoding M4 metalloproteinases), *colA* and *colP* (encoding collagenases) and the seven haemolysin toxins were encoded in the *V. europaeus* core genome. Metalloproteinases VemA and PrtV and their homologues are related to proteolysis and host colonization in a broad range of *Vibrio* taxa, such as *Vibrio cholerae*, *V. anguillarum*, *Vibrio aestuarianus*, *V. coralliilyticus*, *V. neptunius* and *Vibrio splendidus* [68,73]. In a previous study, Martínez [35] determined that a mutant of *V. europaeus* CECT8136 defective in the *vemA* gene resulted in a slightly slower pathogenic process in larvae and juveniles of Manila clam and displayed a chemotaxis ability favoured by VemA to colonize the body mucus of clams and to form biofilm. More recent functional studies further demonstrated that the core protein VemA plays a key role in the pathogenic potential of extracellular proteins towards Manila clam juveniles, as deletion of *vemA* significantly increased host survival to 60%, compared with 22% for the wild-type strain [59]. Together with the virulence assay results presented here, these findings reinforce our hypothesis that the key determinants required for virulence in *V. europaeus* are predominantly encoded within the core genome.

All *V. europaeus* strains were resistant to cephalexin and erythromycin, and they were sensitive to most of the antibiotics tested. Previous studies demonstrated the high prevalence of macrolide- and cephalosporin-resistance genes among *Vibrio* species, including the aquaculture pathogen *V. parahaemolyticus* [74]. In relation to the ARGs, the genetic basis of resistance to cephalosporins has not been identified in *V. europaeus* and could be due to specific mutations [75]. It is important to note that four *V. europaeus* strains isolated from the Spanish Hatchery B were also resistant to two additional antimicrobials. Hatcheries and bivalves are optimal environments to promote the bacterial acquisition of ARGs by HGT due to (i) the extended use of antimicrobials to prevent or control bacterial diseases [76] and (ii) the bioaccumulation of antimicrobials in bivalve tissues [8,77, 78]. This highlights the risk of using antibiotics in shellfish aquaculture, despite most of the antibiotics tested being potentially useful to fight *V. europaeus* [8,77, 78].

From an environmental perspective, secondary metabolites are connected to the capacity of the bacteria to occupy their ecological niche, facilitate access to specific nutrients, compete for resources or establish relationships with other micro-organisms [79,80]. Most of the secondary metabolites produced by *V. europaeus* (NRPS, RiPP-like, betalactone, arylpolyene and ectoine) were found in other marine vibrios [81,82]. A highly conserved amphibactin system was found in *V. europaeus*, and this metabolite, related to the capacity to acquire iron, is widespread in commensal and pathogenic *Vibrio* associated with bivalve haemolymph [83]. The role of ectoine and arylpolyene could be related to bacterial fitness under osmotic and oxidative stress [84,85]. The ecological role of the RiPP (GCF4), the arylpolyene (GCF12) and the PKS-NRP hybrid families (GCF1 and GCF2) must be elucidated due to their high prevalence in the *V. europaeus* strains.

Our results indicate that intra-specific variability in *V. europaeus* is largely driven by the anti-phage defence systems encoded mostly in the accessory genome. This sheds light on the vast and diverse repertoire of anti-phage defence systems encoded by *V. europaeus*. According to Hussain [86], anti-phage protection is cumulative, and those defence systems constitute a large fraction of the accessory genome, accounting for even more than 90% of the accessory genome among closest relatives. This means that phage defence elements can evolve and transfer from cell to cell without interfering with metabolic or physiological processes encoded by the core genome, maintaining the core genome over the long term even in the face of phage predation [86]. The association of anti-phage system genes with the accessory genome reflects the host’s adaptation in the phage-bacteria arms race. This idea is supported by the presence of multiple defence profiles in close temporal and physical space, especially in clonal strains or strains belonging to the same phylogenetic cluster [87,90]. Therefore, the presence of clonal strains harbouring distinct phage defence repertoires within our *V. europaeus* dataset – especially those belonging to phylogenetic subcluster Ia isolated from Hatchery B – supports the view that phage resistance represents one of the major selective forces shaping clonal diversity, as proposed by Hussain [86].

Consistently, a recent study characterizing the mobilome of *V. europaeus* demonstrated that 73% of the anti-phage defence systems identified in the pangenome were associated with mobile genetic elements (MGEs), including plasmids and chromosomal elements, such as prophages, integrative and conjugative/mobilizable elements (ICEs/IME), phage satellites and unclassified chromosomal regions of genomic plasticity. These findings reinforce the role of horizontal gene transfer as a major driver of phage resistance through the acquisition and turnover of MGEs [21]. The repertoire of defence systems is known as the defensome, and its understanding is important when considering the therapeutic use of phages in aquaculture. In this sense, phage therapy could not be effective against *V. europaeus* due to the important and diverse defence arsenal encoded by its accessory genome. Some authors have demonstrated that the rapid acquisition of bacterial resistance against phages offers a parallel to the spread of antibiotic resistance on plasmids in bacteria [86]. Thus, the routine administration of a phage cocktail in shellfish aquaculture to prevent bacterial diseases could not be the optimal approach due to its limited application.

In summary, the pangenome analysis of *V. europaeus* reveals a higher proportion of core genes compared with other pathogenic vibrios, suggesting a conserved virulence arsenal and ecological specialization to shellfish hosts. Moreover, the presence of multiple secretion systems highlights its pathogenic potential, demonstrated in juvenile infection assays. Finally, the high number of anti-phage defence systems encoded in the accessory genome explains almost all the variability of the species. This study presents the first characterization of the pangenome of the bivalve pathogen *V. europaeus*, contributing to an increased understanding of the genomics and ecology of the species.

## Supplementary material

10.1099/mgen.0.001682 Uncited Supplementary Material 1.

## Abbreviations

ADSPs: anti-phage defence system profiles
BGCs: biosynthetic gene clusters
COG: Cluster of Orthologous Group
FSW: filtered seawater
GCFs: gene cluster families
MGEs: mobile genetic elements
NCBI: National Center for Biotechnology Information
NRPS: non-ribosomal peptide synthetases
PCA: principal component analysis
RT: room temperature
VFC: virulence factor classes
