# Supplementary material for: Deciphering the pangenome of the shellfish pathogen Vibrio europaeus: evolutionary history and functional impact of core and accessory genes in aquaculture
Source: Microb Genom. 2026 Jun 4;12(6):001682. doi: 10.1099/mgen.0.001682 (PMC13249269; doi:10.1099/mgen.0.001682)
Supplement: Uncited Supplementary Material 1. [file mgen-12-01682-s001.pdf]

Deciphering the pangenome of the shellfish pathogen *Vibrio europaeus*: Evolutionary history and functional impact of core and accessory genes in aquaculture.

Sergio Rodriguez, Diego Rey-Varela, Andrés Blanco-Hortas, Clara Martinez, Paulino Martínez, Marie-Agnés Travers, Juan L. Barja, Javier Dubert

Supplementary Material

Supplementary Table S1. Genomic information of the *V. europaeus* strains (n= 39) used in this study. Genome completeness and contamination percentages were estimated using CheckM (v1.2.3; Parks et al., 2015). N50 and L50 values were retrieved from the NCBI database. Assembly levels are indicated as follows: scaf (scaffold), compl (fully resolved), and cont (contigs); the number of scaffolds or contigs is shown in brackets. Assemblies marked with an asterisk were generated in previous studies, as indicated in Table 1, and retrieved from NCBI.

Strain	Assembly	CDS	Accession number	Size (Mb)	N50	L50	Completeness (%)	Contamination (%)	GC (%)	Coverage
EX1	comp (4)	4936	CP180205 (ChrI=3.3 Mb)<br>CP180205 (ChrII= 1.7 Mb)<br>CP180207 (pVE-1=330 kb)	5.4	3.3 Mb	1	99.64	0.58	45	150x
PP-654	scaf (38)	4921	JAPFJT000000000.1	5.4	813.1 kb	3	99.9	0.8	45	241x
PP-660	scaf (34)	4891	JAPFJS000000000.1	5.4	813.1 kb	3	99.9	0.8	45	215x
PP-635	scaf (38)	5014	JAPFJR000000000.1	5.6	635.1 kb	4	99.96	2.11	45	217x
CECT8136T=PP-638	comp (4)	4936	LUAX00000000.1	5.5	3.3 Mb	1	98.78	0.71	45	105x
CECT8427	comp (4)	4941	JAPFJQ010000001.1 (ChrI=3.3 Mb)<br>JAPFJQ010000004.1 (ChrII=1.8 Mb)<br>JAPFJQ010000003.1 (pVeu-1= 362 kb)<br>JAPFJQ010000002.1 (pVeu-3=71 kb)	5.6	3.3 Mb	1	98.73	0.88	45	191x
CECT8426=07/118 T2	comp (3)*	4935	CP064858.1 (ChrI=3.3 Mb)<br>CP064857.1 (ChrII=1.8 Mb)<br>CP064859.1 (pVeu-1=419.2 kb)	5.6	3.3 Mb	1	99.06	1.17%	45	118x
07/038 2T2	scaf (44)	5008	JAPFJP000000000.1	5.5	494.1 kb	4	100	0.88	45	216x
07/108 T1	scaf (43)	4887	JAPFJO000000000.1	5.4	656 kb	4	99.83	0.85	45	181
07/110 T1	scaf (43)	4880	JAPFJN000000000.1	5.5	1.1 Mb	2	100	1.12	45	259x
07/112 T1	scaf (36)	4878	JAPFJM000000000.1	5.5	1.1 Mb	2	100	1.12	45	210x
07/115 T2	scaf (47)	4856	JAPFJL000000000.1	5.3	584.7	3	99.83	0.82	44.5	213x
07/116 T1	scaf (37)	4851	JAPFJK000000000.1	5.4	723.5 KB	3	100	1.12	45	229x
07/117 T1	scaf (37)	4908	JAPFJJ000000000.1	5.5	389.5 kb	4	99.87	0.92	45	221x
07/120 T1	scaf (41)	4998	JAPFJI000000000.1	5.5	645.3 kb	4	99.61	1.26	45	245x
07/121 1T1	scaf (39)	4883	JAPFJH000000000.1	5.5	1.5 Mb	2	100	1.12	45	214x
PP2-843	comp (4)	5086	JAPFJG010000002.1 (ChrI=3.4 Mb)<br>JAPFJG010000004.1 (ChrII=1.7 Mb)<br>JAPFJG010000001.1 (pVeu-1=389 kb)	5.7	3.4 Mb	1	98.78	0.71	45	233x

			JAPFJG010000003.1 (Unknown contig=77 kb)							
PP2-978	scaf (32)	5015	JAPFJF000000000.1	5.5	813.1 kb	3	99.83	0.58	45	219x
2909	scaf (34)	5019	JAPFJE000000000.1	5.5	435.7 kb	4	99.83	0.58	45	206x
2895	scaf (33)	4884	JAPFJD000000000.1	5.4	1 Mb	2	99.96	0.83	45	209x
2930	scaf (36)	4952	JAPFJC000000000.1	5.5	435.6 kb	4	99.96	0.83	45	208x
2951	scaf (37)	4944	JAPFJB000000000.1	5.5	697.9 kb	3	99.96	0.92	45	204x
2945	scaf (33)	4844	JAPFJA000000000.1	5.4	1.2	2	99.96	0.83	45	205x
2967	scaf (33)	5015	JAPFIZ000000000.1	5.5	813 kb	3	99.83	0.58	45	193x
2968	scaf (34)	4940	JAPFIY000000000.1	5.5	781.3 kb	3	99.96	0.92	45	194x
2969	scaf (37)	4941	JAPFIX000000000.1	5.5	1 Mb	2	99.96	0.92	45	204x
2971	scaf (35)	4943	JAPFIW000000000.1	5.5	1.1	2	99.96	0.92	45	228x
2974	scaf (39)	5124	JAPFIV000000000.1	5.6	435.6 kb	4	99.83	0.58	45	207x
2975	scaf (35)	4941	JAPFIU000000000.1	5.5	1 Mb	2	99.96	0.92	45	215x
3454	scaf (45)	5205	JAPFIT000000000.1	5.7	329.6 kb	5	99.96	1.05	45	195x
3492	scaf (45)	5203	JAPFIS000000000.1	5.7	651.7 kb	4	99.96	1.05	45	244x
3610	scaf (40)	5199	JAPFIR000000000.1	5.7	456.8 kb	4	99.96	1.05	45	202x
3614	scaf (40)	5129	JAPFIQ000000000.1	5.7	781.4	3	99.96	1.05	45	260x
NPI1	comp (3)*	4811	CP053541.1 (ChrI=3.3 Mb)<br>CP053543.1 (ChrII=1.8 Mb)<br>CP053542.1 (pVeu-1=336 kb)	5.4	3.3 Mb	1	98.5	1.02	45	276x
071316F	cont (85)*	4923	VTYH00000000.1	5.5	212 kb	9	99.74	0.85	45	50x
L2	scaf (40)	5049	JAPFIP000000000.1	5.5	768.4 kb	3	99.83	0.58	45	247x
L3	scaf (40)	5011	JAPFIO000000000.1	5.5	435.9 kb	4	99.96	0.97	45	238x
L4	scaf (36)	4881	JAPFIN000000000.1	5.4	679.3 kb	3	99.96	0.97	45	245x
L20	scaf (37)	5011	JAPFIM000000000.1	5.5	435.8	4	99.96	0.97	45	221x

Supplementary Table S2. tRNAs found from the *V. europaeus* genomes used in this study. Strains with fully resolved genomes are marked with asterisks.

Strain	tRNAs decoding Standard 20 AA	tRNAs with undetermined/unknown isotypes	Predicted pseudogenes	Total tRNAs
07/038 2T2	101	2	3	106
07/108 T1	100	1	0	101
07/110 T1	106	1	0	107
07/112 T1	106	0	0	106
07/115 T2	107	0	1	108
07/116 T1	107	0	0	107
07/117 T1	102	1	1	104
07/120 T1	101	0	2	103
07/121 1T1	108	1	0	109
071316F	85	0	3	88
2895	104	2	0	106
2909	97	1	2	100
2930	97	3	1	101
2945	98	2	0	100
2951	100	3	0	103
2967	100	1	1	102
2968	101	3	0	104
2969	101	2	0	103
2971	105	3	1	109
2974	97	1	1	99
2975	101	3	0	104
3454	95	2	0	97
3492	102	3	0	105
3610	99	3	0	102
3614	102	3	0	105
CECT8136T*	116	2	0	118
CECT8426*	119	0	2	121
CECT8427*	121	0	1	122
EX1*	118	0	1	119
L2	101	2	1	104
L20	97	3	0	100
L3	100	3	1	104
L4	103	3	0	106
NPI-1*	116	1	1	118
PP2-843*	119	1	1	121
PP2-978	102	1	2	105
PP-635	100	3	0	103
PP-654	100	2	0	102
PP-660	100	3	0	103

Supplementary Table S3. rRNAs found from the *V. europaeus* genomes used in this study. Strains with fully resolved genomes are marked with asterisks.

Strain	16S rRNA	5S rRNA	23S rRNA
07/038 2T2	1	2	1
07/108 T1	1	3	1
07/110 T1	1	3	1
07/112 T1	1	3	1
07/115 T2	1	1	1
07/116 T1	1	3	1
07/117 T1	1	2	1
07/120 T1	1	1	1
07/121 1T1	1	2	1
071316F	1	1	1
2895	1	2	1
2909	1	3	1
2930	1	2	1
2945	1	2	1
2951	1	3	1
2967	1	2	1
2968	1	3	1
2969	1	2	1
2971	1	2	1
2974	1	2	1
2975	1	4	2
3454	1	2	1
3492	1	2	1
3610	1	2	1
3614	1	2	1
CECT8427*	10	11	10
CECT8136T*	9	10	9
CECT8426*	10	11	10
EX1*	10	11	10
L2	1	2	1
L20	1	3	1
L3	1	2	1
L4	1	2	1
NPI-1*	9	10	9
PP2-843*	10	10	10
PP2-978	1	3	1
PP-635	1	2	1
PP-654	1	3	1
PP-660	1	2	1

Supplementary Table S4. Virulence factors identified from the *V. europaeus* pangenome.
Core virulence-related genes are indicated in bold. Virulence factor classes (VFCs) are numbered according to Fig. 4.

VFclass	Virulence factors	Related genes
Adherence (VFC 2)	Mannose-sensitive hemagglutinin (MSHA type IV pilus)	*mshA, mshB, mshC, mshE, mshG, mshH, mshI, mshJ, mshK, mshL, mshM, mshN.*
	Type IV pilus	*pilA, pilB, pilC, pilD.*
	Curli fibers(*Escherichia*)	***csgG***
	LPS-O-antigen (P. aeruginosa)(*Pseudomonas*)	*tviB, tviB*(2), *hisF2.*
	Tap type IV pili(*Aeromonas*)	*tapQ*
	The tad locus(*Haemophilus*)	***tadA*, *tadA*(2)**
	Type IV pili(*Yersinia*)	*pilW*
Antiphagocytosis (VFC 3)	Capsular polysaccharide	*cpsA, cpsC, rmlA, rmlB, rmlC, rmlD, wbfT, wbfU, wbfV/wcvB, wbfY.*
	Capsule (*Klebsiella*)	*uge*
Chemotaxis and motility (VFC 5)	Flagella	***cheA*, *cheA*(2), *cheB*, *cheR*, *cheV*, *cheW*, *cheY*, *cheZ*, *filM*, *flaA*, *flaB*, *flaC*, *flaD*, *flaE*, *flaG*, *flaI*, *flgA*, *flgB*, *flgC*, *flgD*, *flgE*, *flgF*, *flgG*, *flgH*, *flgI*, *flgJ*, *flgK*, *flgL*, *flgM*, *flgN*, *flhA*, *flhB*, *flhF*, *flhG*, *fliA*, *fliD*, *fliE*, *fliF*, *fliG*, *fliH*, *fliI*, *fliJ***, *fliK*, ***fliL*, *fliN*, *fliO*, *fliP*, *fliQ*, *fliR*, *fliS*, *flrA*, *flrB*, *flrC*, *motA*, *motB*, *motX*, *motY*.**
Enzyme (VFC 7)	Metalloproteinase	***VemA*, *prtV***
	Collagenase	***colA*, *colP***
Iron uptake (VFC 10)	Enterobactin receptors	***vctA***
	Heme receptors	***hutA***
	Periplasmic binding protein-dependent ABC transport systems	*vctC, vctD, vctG, vctP, vctP*(2)
	Pyoverdine(*Pseudomonas*)	*pvdY*
Quorum sensing (VFC 13)	Autoinducer-2	***luxS***
	Cholerae autoinducer-1	***cqsA***
Secretion system (VFC 14)	EPS T2SS	***epsC, epsE, epsF, epsG, epsH, epsI, epsJ, epsK, epsL, epsM, gspD***
	T3SS1 secreted effectors	Undetermined, *vopQ*
	T3SS1	*sycN, tyeA, vcrD, vcrG, vcrH, vcrR, virF, virG, vopB, vopN, vscA, vscB, vscC, vscD, vscF, vscG, vscI, vscJ, vscK, vscL, vscN, vscO, vscQ, vscR, vscS, vscT, vscU, vscX, vscY, vxsC.*
	VAS effector proteins	*hcp-2, hcp-2*(2), *vgrG*-2, *vgrG*-2*(2), vgrG*-2(3), *vgrG*-3

	VAS T6SS secretion system	***vasA*, *vasB*, *vasC*, *vasD*, *vasE*, *vasF*, *vasG*, *vasH*, *vasJ*, *vasK*, *tssE*, *impC/vipB*, *ipmB/vipA***, *impA*, ***impA*(2)**, *hcp*, *hcp*(2), ***hcp*(3)**, *impB/vipA*, ***impB/vipA*(2)**, *impC/vipB*, ***impC/vipB*(2)**, ***impC/vipB*(3)**, *tssE*, ***tssE*(2)**, *impG/vasA*, ***impG/vasA*(2)**, *impH/vasB*, ***impH/vasB*(2)**, *tssH*, ***tssH*(2)**, *impI/vasC*, ***impI/vasC*(2)**, *vasD*, ***vasD*(2)**, *impJ/vasE*, ***impJ/vasE*(2)**, *impK/vasF/ompA/motB*, ***impK/vasF/ompA/motB* (2)**, *icmF/vasK*, ***icmF/vasK*(2)**, PAAR-repeat_protein, **PAAR-repeat_protein(2)**.
	T3SS(*Aeromonas*)	*ati1*
Toxin (VFC 15)	RTX toxin	*rtxB, rtxD*
	V.cholerae cytolysin	*hlyA*
	V.cholerae cytolysin	*hlyA*
	V.cholerae cytolysin	*hlyA*
	V.cholerae cytolysin	*hlyA*
	V.cholerae cytolysin	*hlyA*
Acid resistance (VFC 1)	Urease(*Helicobacter*)	***ureB*, *ureG***
Biofilm formation (VFC 4)	AdeFGH efflux pump/transport autoinducer(*Acinetobacter*)	***adeG***
Endotoxin (VFC 6)	LOS(*Haemophilus*)	*kdsA, kpsF*
	LPS(*Bordetella*)	*bplA*
Glycosylation system (VFC 8)	O-linked flagellar glycosylation(*Campylobacter*)	*pseB*
Immune evasion (VFC 9)	Capsule(*Neisseria*)	*ctrD*
	Capsule(*Acinetobacter*)	*pseI, pseF, pseC, wbpD, tviB(3), pseB(2), vipB/tviC, wbpP*
Nutritional factors (VFC 11)	Allantoin utilization(*Klebsiella*)	*allA*
Others (VFC 12)	O-antigen(*Yersinia*)	*manA, wcaG*

Supplementary Table S5. Differences in the non-core virulence factors encoded by the strains belonging to the same phylogenetic cluster.

Strains encoding a certain gene are indicated in bold.

**Subcluster Ia**	immune evasion gene *ctrD* (**L4**)
**Subcluster Ib**	VAS effector protein coding genes *vgrG-2* (**2967, 2974** and **2909**) and *vgrG-2_3* (**PP2-978**); toxins coding genes *cysC1* (**2967, 2974** and **PP2-978**) or *rtxE* (**2909**); allantoin utilization gene *allA* (**2967**).<br>**L2:** VAS effector proteins of the T6SS (*hcp2_2, vgrG-2, vgrG-2_3* and *vgrG-3*), toxins (*rtxB, rtxD, rtxE, hlyA* and *cysC1*), endotoxins (*kdsA* and *kpsF*), immune evasion (*ctrD*) and allantoin utilization (*allA*).
**Subcluster IIa**	No differences
**Subcluster IIb**	type 4 pili *tapQ* and the toxin *cysC1* (**07/120 T1**); *rtxE* and *allA* (**CECT8426**).
**Cluster III**	type IV pili *pilW* and VAS effectors *hcp-2_2* and *vgrG2_3* (**07/117 T1**).

Supplementary Table S6. Consensus antimicrobial resistance phenotypes identified in the *V. europaeus* pangenome.

The table includes information on antimicrobial resistance phenotypes obtained using the disc-diffusion assay (antibiotics and concentrations are indicated in brackets), as well as antibiotic resistance genes identified using the CARD and ResFinder databases. Discrepancies between in silico predictions and phenotypic results are also indicated; in such cases, the experimental disc-diffusion result was used as the consensus phenotype. All strains were sensitive to chemicals used as disinfectants such as quaternary ammonium compounds (cetylpyridinium chloride, chlorhexidine, benzalkoniumchloride and ethidium bromide), aldehydes (formaldehyde) and peroxides (hydrogen peroxide). In addition, all *V. europaeus* strains were sensitive to the following antibiotic classes: rifamycin (rifampicin), cephalosporin (ceftiofur), beta-lactam (piperacillin+clavulanic acid, cefotaxime, piperacillin, cefixime, ampicillin, cefotaxime+clavulanic acid, ticarcillin, amoxicillin+clavulanic acid, ertapenem, cefepime, ceftriaxone, ceftazidime+avibactam, cephalotin, unknown beta-lactam, ampicillin+clavulanic acid, ticarcillin+clavulanic acid, temocillin, meropenem, imipenem, penicillin, piperacillin+tazobactam, cefoxitin, ceftazidime, amoxicillin, aztreonam), polymyxin (colistin), quinolone (ciprofloxacin, nalidixic acid, unknown quinolone, fluoroquinolone), , amphenicol (chloramphenicol), streptogramin A (dalfopristin, pristinamycin IIa, virginiamycin M, quinupristin+dalfopristin), folate pathway antagonist (sulfamethoxazole, trimethoprim), pleuromutilin (tiamulin), glycopeptide (vancomycin, teicoplanin), steroid antibacterial (fusidic acid), macrolide (oleandomycin, carbomycin, telithromycin, azithromycin, tylosin, , spiramycin), streptogramin B (quinupristin, virginiamycin S, pristinamycin IA), oxazolidinone (linezolid), aminoglycoside (kasugamycin, apramycin, gentamicin, arbekacin, tobramycin, butiromycin, bleomycin, dibekacin, unknown aminoglycoside, paromomycin, butirosin, kanamycin, sisomicin, astromicin, ribostamycin, fortimicin, amikacin, isepamicin, hygromycin, netilmicin, neomycin, lividomycin), fosfomycin (fosfomycin), nitroimidazole (metronidazole), aminocyclitol (spectinomycin), pseudomonic acid (mupirocin), tetracycline (tigecycline, tetracycline, doxycycline, minocycline), and lincosamide (clindamycin, lincomycin).

**Class**	**Antimicrobial**	**Strains**	**Resistant by disk diffusion assay**	**ARGs**	**Discrepancies**
Macrolide	erythromycin	All	Yes (E15)	*crp*	No
Aminoglycoside	streptomycin	3454, 3492, 3610 and 3614	Yes (S10)	*aph(3'')-Ib* and *aph(6)-Id*	No
Synthetic antibiotics	sulfonamide	3454, 3492, 3610 and 3614	Yes (SULDD25)	*sul2*	No
Cephalosporin	cephalexin	All	Yes (CN30)	*No*	Yes
Amphenicol	Florfenicol	3454, 3492, 3610 and 3614	No (FFC30)	*floR*	Yes
tetracycline	tetracycline	All	No (TE30)		

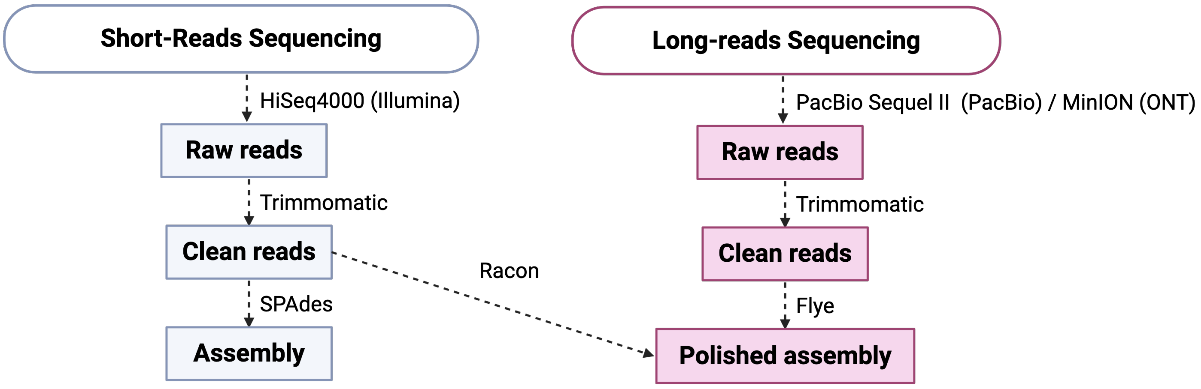

Supplementary Figure S1. Workflow of genome sequencing and assembly.
The workflow illustrates the main analytical steps included in each analysis (pipelines used are also shown):
Short-read sequencing: 150 bp paired-end Illumina sequencing was performed in the V. europaeus strains (36 strains) for assembling at the scaffold level (Table 1). Strains were grown overnight in Trypto–Casein Soy agar supplemented with 2% (w/v) sodium chloride (TSA–2, Condalab) at 25ºC. Subsequently, a single colony was picked and grown under the same bacterial culture conditions in broth (TSB-2) with vigorous shaking. DNA was extracted from an aliquot of the overnight culture (200 µl) using the DNeasy Blood & Tissue Kit (QIAGEN) following the manufacturer instructions. Quantity, quality and integrity of each DNA extraction were evaluated using NanoDrop One (Thermo Scientific), Qubit (Thermo Scientific) and by electrophoresis in a 1% (w/v) agarose gel. Genomic libraries and sequencing (HiSeq4000 sequencer, Illumina) were performed by the SNPsaurus company. Quality control of Illumina reads was performed using Trimmomatic (Bolger, 2014) and paired-end short reads were assembled at the scaffold level using SPAdes 3.15.4 (Prjibelski, 2020).
Long-read sequencing: Four representative strains (the type strain CECT8136 and the isolates CECT8427, PP2-843, and EX1; Table 1) were long-read sequenced to achieve high-resolution assemblies polished with Illumina reads from the previous section. For this, each bacterial strain was grown as described above and HMW DNA was extracted from 4 ml of an overnight culture using the Qiagen Genomic-tips 100/G kit (QIAGEN). Quantity, quality, and integrity of HMW DNA extractions were evaluated as described above. CECT8136, CECT8427, and PP2-843 strains were sequenced using a PacBio Sequel II sequencer (PacBio) by SNPsaurus, while yhe EX1 genome was sequenced using a MinION sequencer with the Rapid sequencing gDNA-barcoding kit (ONT). Assemblies from PacBio and ONT reads were performed using the de novo long-read assembler Flye (Kolmogorov, 2019) and polished by Racon (Vaser, 2017). Finally, each assembly was polished using Illumina reads with Pilon (Walker, 2014). Thus, a total of six whole-genome chromosome-level assemblies (CECT8136T, CECT8427, PP2-843, and EX1 sequenced in this study; and NPI-1 and CECT8426 retrieved from NCBI) were used in this study for pangenome analyses (Table 1).
56

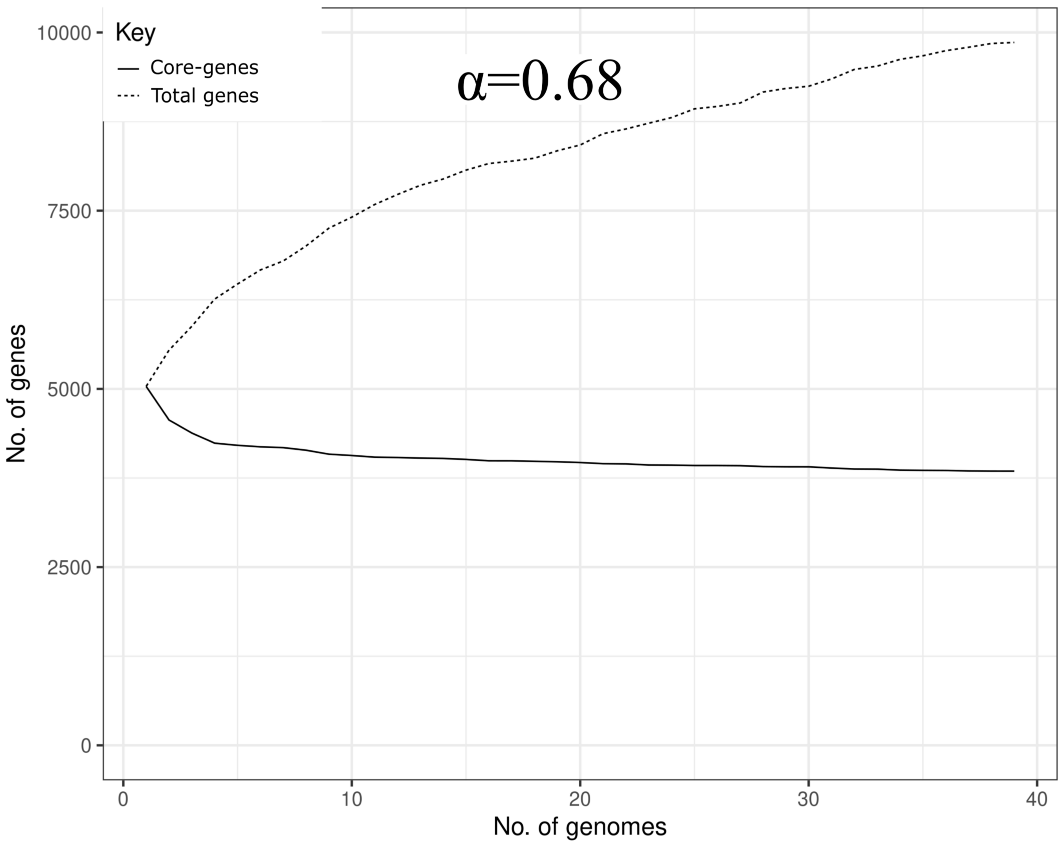

Supplementary Figure S2. Relation between the number of genes (core and total) and the number of genomes.
The α coefficient (value=0.68) of Heap's Law for the conserved curve is shown.

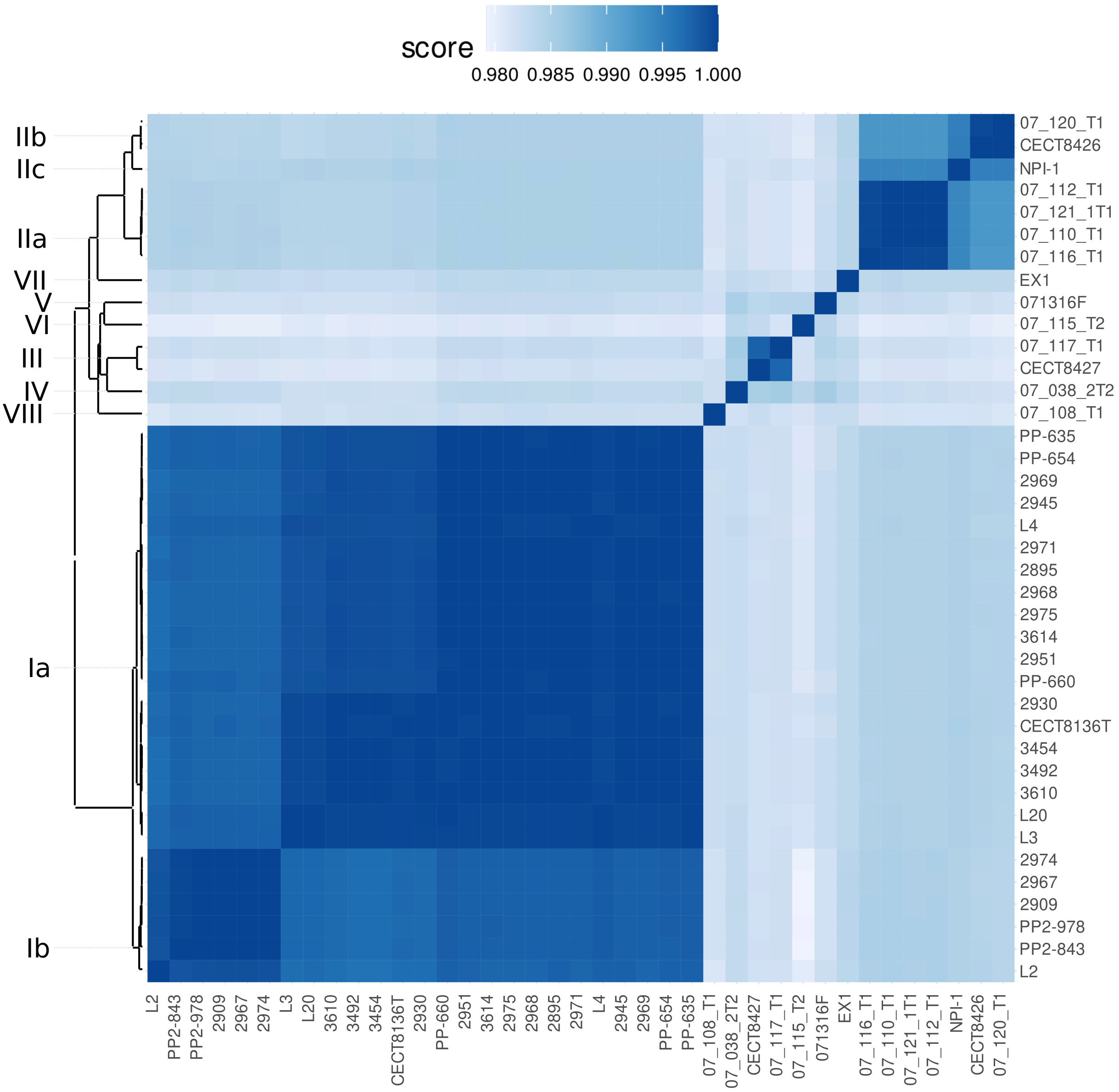

Supplementary Figure S3. ANI heatmap of *V. europaeus* strains with hierarchical clustering.

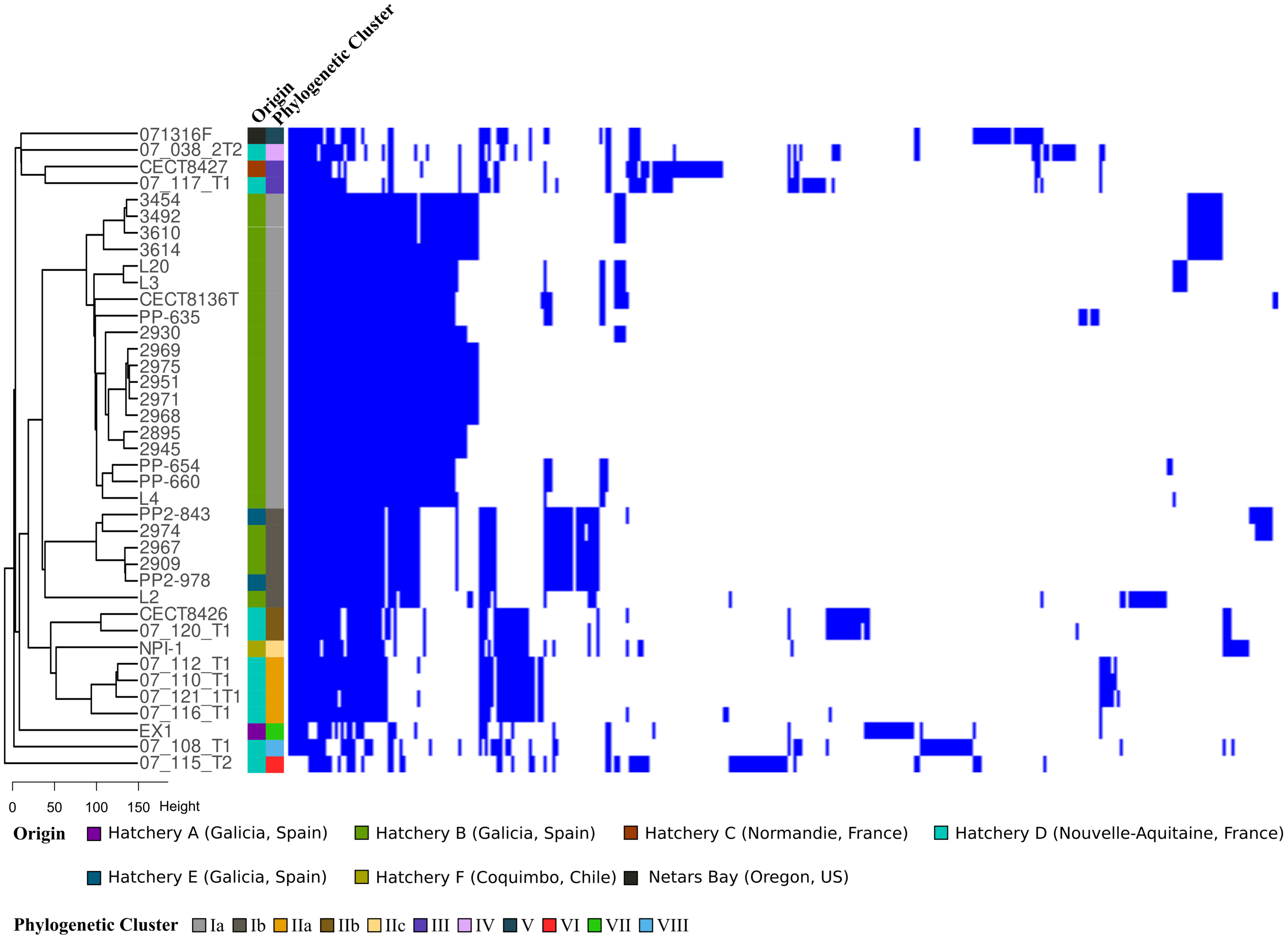

Supplementary Figure S4. Presence/absence matrix showing the *V. europaeus* accessory genes. Strains are sorted according to the dendrogram plotted on the left.

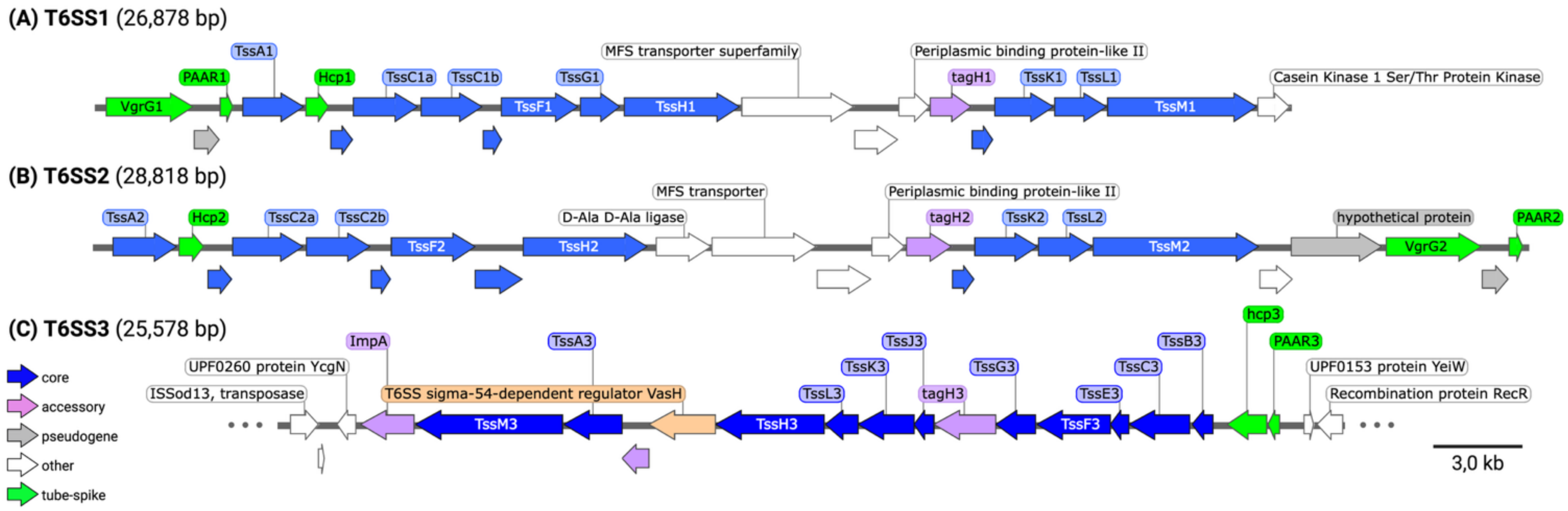

Supplementary Figure S5. T6SS (T6SS1, A; T6SS2, B; T6SS3, C) gene clusters found in *V. europaeus* genomes. T6SS1 and T6SS3 were assigned to the core genome.

T6SS genes are denoted by arrows indicating the predicted direction of the transcription. Encoded proteins or domains are denoted on the genes.

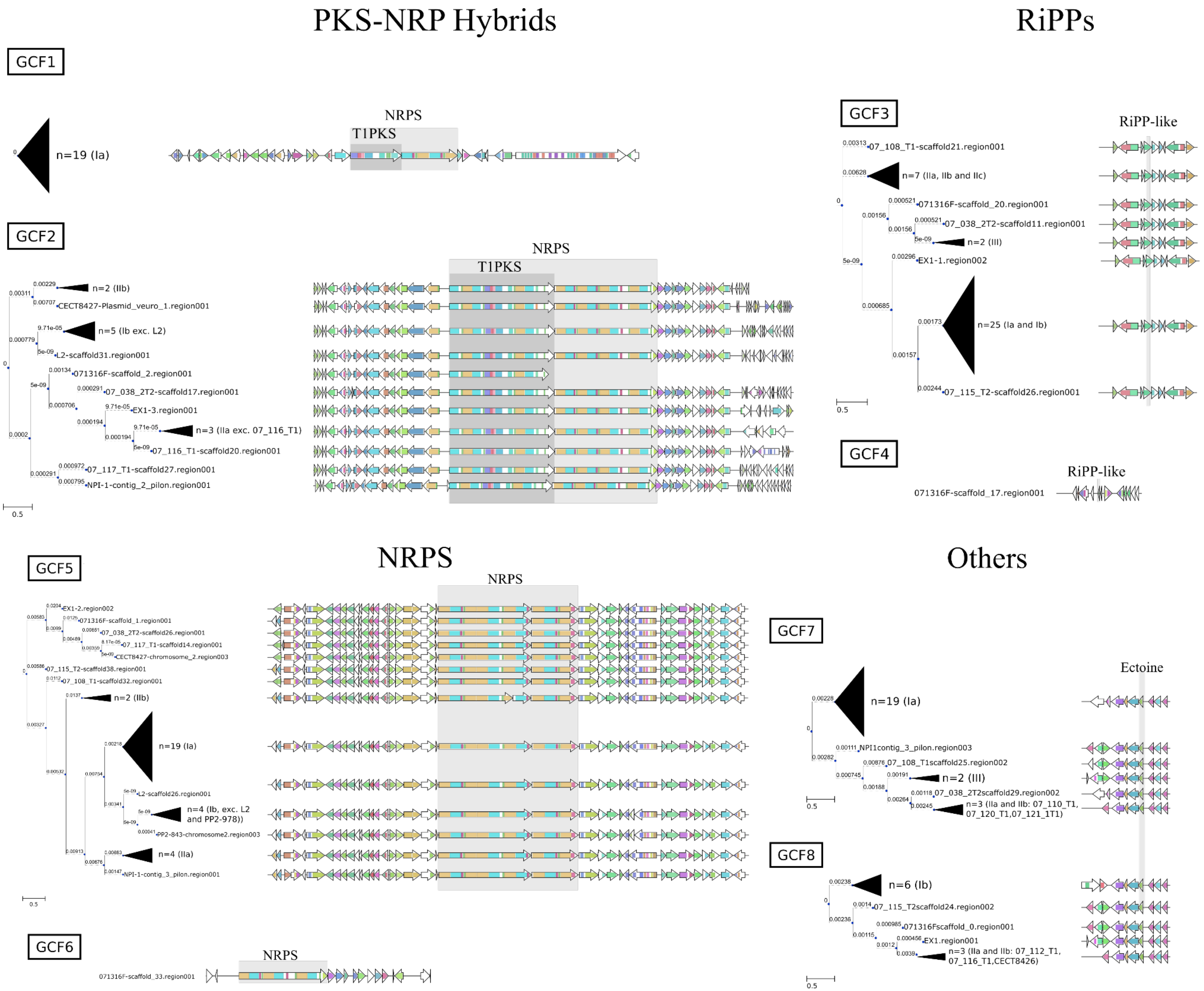

PKS-NRP Hybrids
RiPPs
GCF1
NRPS
T1PKS
n=19 (Ia)
GCF2
n=2 (IIb)
CECT8427-Plasmid_veuro_1.region001
n=5 (Ib exc. L2)
L2-scaffold31.region001
071316F-scaffold_2.region001
07_038_2T2-scaffold17.region001
EX1-3.region001
n=3 (IIa exc. 07_116_T1)
07_116_T1-scaffold20.region001
07_117_T1-scaffold27.region001
NPI-1-contig_2_pilon.region001
0.5
GCF3
RiPP-like
07_108_T1-scaffold21.region001
n=7 (IIa, IIb and IIc)
071316F-scaffold_20.region001
07_038_2T2-scaffold11.region001
n=2 (III)
EX1-1.region002
n=25 (Ia and Ib)
07_115_T2-scaffold26.region001
0.5
GCF4
RiPP-like
071316F-scaffold_17.region001
NRPS
Others
GCF5
EX1-2.region002
071316F-scaffold_1.region001
07_038_2T2-scaffold26.region001
07_117_T1-scaffold14.region001
CECT8427-chromosome_2.region003
07_115_T2-scaffold38.region001
07_108_T1-scaffold32.region001
n=2 (IIb)
n=19 (Ia)
L2-scaffold26.region001
n=4 (Ib, exc. L2 and PP2-978))
PP2-843-chromosome2.region003
n=4 (IIa)
NPI-1-contig_3_pilon.region001
0.5
GCF6
NRPS
071316F-scaffold_33.region001
GCF7
Ectoine
n=19 (Ia)
NPI1contig_3_pilon.region003
07_108_T1scaffold25.region002
n=2 (III)
07_038_2T2scaffold29.region002
n=3 (IIa and IIb: 07_110_T1, 07_120_T1,07_121_1T1)
0.5
GCF8
n=6 (Ib)
07_115_T2scaffold24.region002
071316Fscaffold_0.region001
EX1.region001
n=3 (IIa and IIb: 07_112_T1, 07_116_T1,CECT8426)
0.5

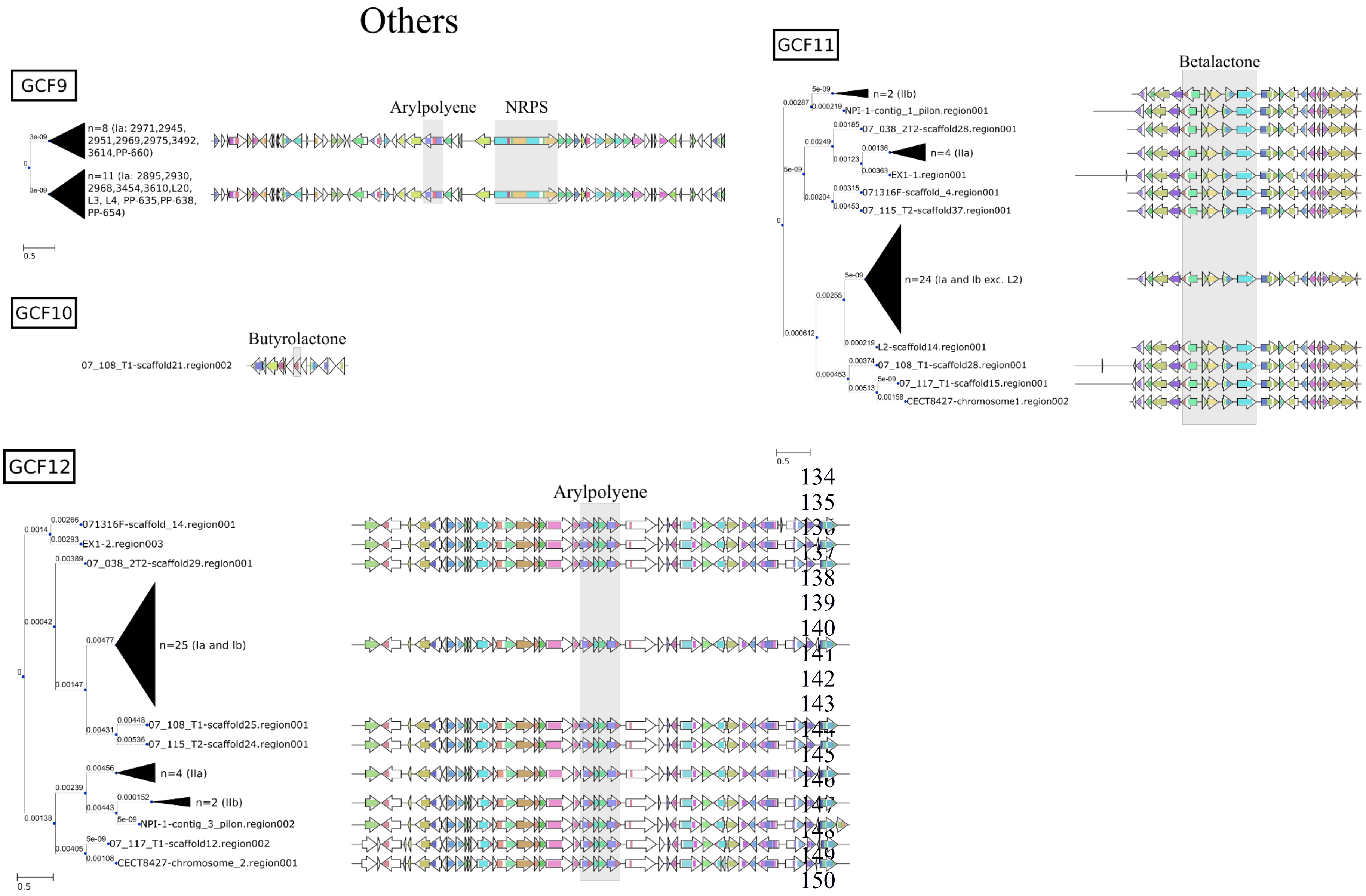

Supplementary Figure S6. Diversity of the 12 biosynthetic gene cluster families (GCF1–GCF12) encoded by *Vibrio europaeus* strains. Branches composed of identical sequences were collapsed. Gene cluster schematics generated with antiSMASH are shown alongside their corresponding branches, and core biosynthetic genes are shaded in grey.
